# Unveiling the Fluorination Pathway of Ruddlesden–Popper Oxyfluorides: A Comprehensive *In Situ* X-ray and Neutron Diffraction Study

**DOI:** 10.1021/jacs.4c18187

**Published:** 2025-02-17

**Authors:** Jonas Jacobs, Andy Bivour, Vadim Sikolenko, Holger Kohlmann, Thomas C. Hansen, James R. Hester, Ke Xu, Jörn Schmedt auf der Günne, Stefan G. Ebbinghaus

**Affiliations:** † Martin Luther University Halle-Wittenberg, Faculty of Natural Sciences II, Institute of Chemistry, Inorganic Chemistry, Kurt-Mothes-Straße 2, 06120 Halle, Germany; ‡ Leipzig University, Institute for Inorganic Chemistry and Crystallography, Johannisallee 29, 04103 Leipzig, Germany; § Institut Laue-Langevin, 71 avenue des Martyrs, 38000 Grenoble, France; ∥ Australian Center for Neutron Scattering, Australian Nuclear Science and Technology Organisation (ANSTO), Locked Bag 2001, Kirrawee DC, New South Wales 2232, Australia; ⊥ University of Siegen, Faculty IV: School of Science and Technology, Department of Chemistry and Biology, Inorganic Materials Chemistry, Adolf-Reichwein-Str. 2, 57076 Siegen, Germany

## Abstract

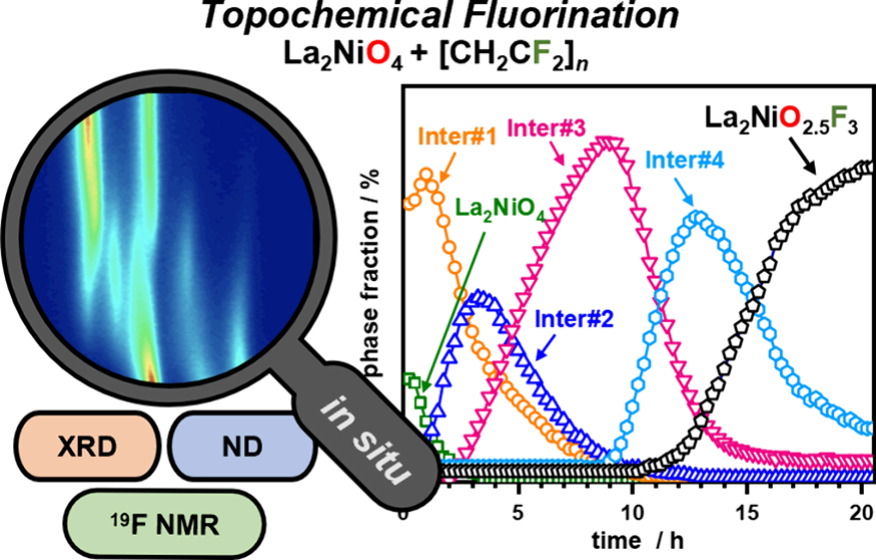

Ruddlesden–Popper oxyfluorides exhibit unique properties, but their synthesis is often hindered by low thermodynamic stability. To overcome this challenge, understanding the formation mechanism of these materials is crucial for optimizing the reaction conditions and accessing new products. This study presents an in-depth investigation of the fluorination reaction of La_2_NiO_4_ with poly(vinylidene fluoride) (PVDF), targeting the oxyfluorides La_2_NiO_3_F_2_ and La_2_NiO_2.5_F_3_, which exhibit distinct structural distortions. *In situ* X-ray diffraction experiments, performed on a laboratory diffractometer, revealed the presence of four distinct reaction intermediates. The crystal structures of these intermediates were further elucidated through X-ray and neutron powder diffraction experiments, complemented by *in situ* neutron powder diffraction data obtained using a setup featuring a low-background cell made from single-crystalline sapphire. ^19^F MAS NMR spectroscopy was employed to localize the fluoride ions and to track the consumption of PVDF. By systematically optimizing reaction conditions, we successfully obtained both oxyfluorides and quantified the phase evolution of all intermediates through extensive Rietveld refinements, yielding the following reaction steps: La_2_NiO_4_ (*I*4/*mmm*) → Inter#1 (*Fmmm*) → Inter#2 (*Fmmm*, with increased orthorhombic distortion) → Inter#3 (*C*2/*c*) → La_2_NiO_3_F_2_ (*Cccm*). In the presence of 50% excess PVDF, La_2_NiO_3_F_2_ is not obtained from Inter#3 and the reaction instead progresses via Inter#4 (*P*4_2_/*nnm*) to La_2_NiO_2.5_F_3_ (*P*4_2_/*nnm*, with a larger unit cell). This study demonstrates the power of laboratory *in situ* XRD experiments in elucidating complex fluorination reaction mechanisms, enabling the synthesis of new oxyfluorides with interesting physical properties. The *in situ* approach represents a significant advancement over traditional trial-and-error methods, which are still prevalent in solid-state synthesis.

## Introduction

The synthesis of new Ruddlesden–Popper (RP) oxyfluoride compounds with layered perovskite structure for application in fluoride ion batteries (FIB)^1,2^ is currently a hot topic in solid state and materials chemistry due to the strong demand for new energy storage materials. Besides the potential application in FIBs, the modification of physical properties through the substitution of O^2–^/F^–^ is another driving force, which strongly promotes the interest in new oxyfluorides. A prominent example is the observation of superconductivity in Sr_2_CuO_2_F_2+*d*_^3−6^ which was first reported in 1995. Other oxyfluorides are currently investigated with respect to superconductivity. Recently, La_2_NiO_3_F^7^ and Pr_2_NiO_3_F^8^ with single-layer T′-structure have gained attention, along with related lanthanide nickelate oxides such as LaNiO_2_ and NdNiO_2_,^9−11^ due to their potential to exhibit nickel in the +1^12^ oxidation state, rendering them isoelectronic to the well-known cuprate superconductors. The T′-nickelate oxyfluorides were obtained by a reductive defluorination reaction of La_2_NiO_3_F_2_ with NaH^7^ or Pr_2_NiO_3_F_2_ with CaH_2_.^8^ Further modifications of physical properties that were recently reported are, for example, the strong increase of the Néel temperature *T*_N_ from ∼50 K for La_0.5_Sr_3.5_Fe_3_O_10-δ_ to >450 K in antiferromagnetic La_0.5_Sr_3.5_Fe_3_O_7.5_F_2.6_^13^ and the increase of the band gap energy from 1.3 eV in La_2_NiO_4_ to 3.4 eV in La_2_NiO_2.5_F_3_^14^ and from 2.3 eV in LaBaInO_4_ to 2.7 eV/3.5 eV in LaBaInO_3_F_2._^15^

The compounds mentioned above were mostly prepared by the topochemical fluorination reaction of the corresponding oxides with the fluorinated polymer poly(vinylidene fluoride) (PVDF). The use of fluorinated polymers, which also includes polytetrafluoroethylene (PTFE), as fluorine sources was first reported for PVDF by Slater in 2002.^16^ Three main advantages over the use of traditional fluorine sources like F_2_ or transition metal difluorides, e.g., CuF_2_, are of importance: (1) Fluorinated polymers are solid and stable at room temperature, which eases handling, and the desired stoichiometries can easily be adjusted. (2) Thermal decomposition of these polymers results in volatile byproducts like CO, CO_2_, HF, and H_2_O, yielding highly pure products without inorganic impurities, which are typically found when transition metal fluorides are used as F^–^ source. (3) The fluorination reaction has a neutral or slightly reductive character unlike the oxidative reaction with F_2_ gas. This enables the accessibility of new oxyfluorides containing less redox stable transition metal cations like Ni, Co, or Fe. Additionally, both polymers exhibit a high limiting oxygen index (LOI) (PVDF > 40%, PTFE > 95%). Heating them in atmospheric air therefore does not result in a self-contained combustion, making the fluorination process controllable in terms of reaction rate.

Even though numerous oxyfluorides (Sr_2_TiO_3_F_2_,^16^ Sr_3_Ti_2_O_5_F_4_,^17^ La_2_Ni_1–*x*_Cu*_x_*O_3_F_2_,^18^ and Sr_2_Ir(O,F)_6−δ_,^19^ to name a few) were prepared by the reaction with PVDF, little is known about the reaction mechanism of the fluorination reactions, and oxyfluoride synthesis almost always consists of numerous experiments applying different temperatures, dwell times, and varying oxide:polymer ratios, which often impedes the observation and isolation of new metastable compounds as they may decompose while the reaction progresses.

**Figure 1:**
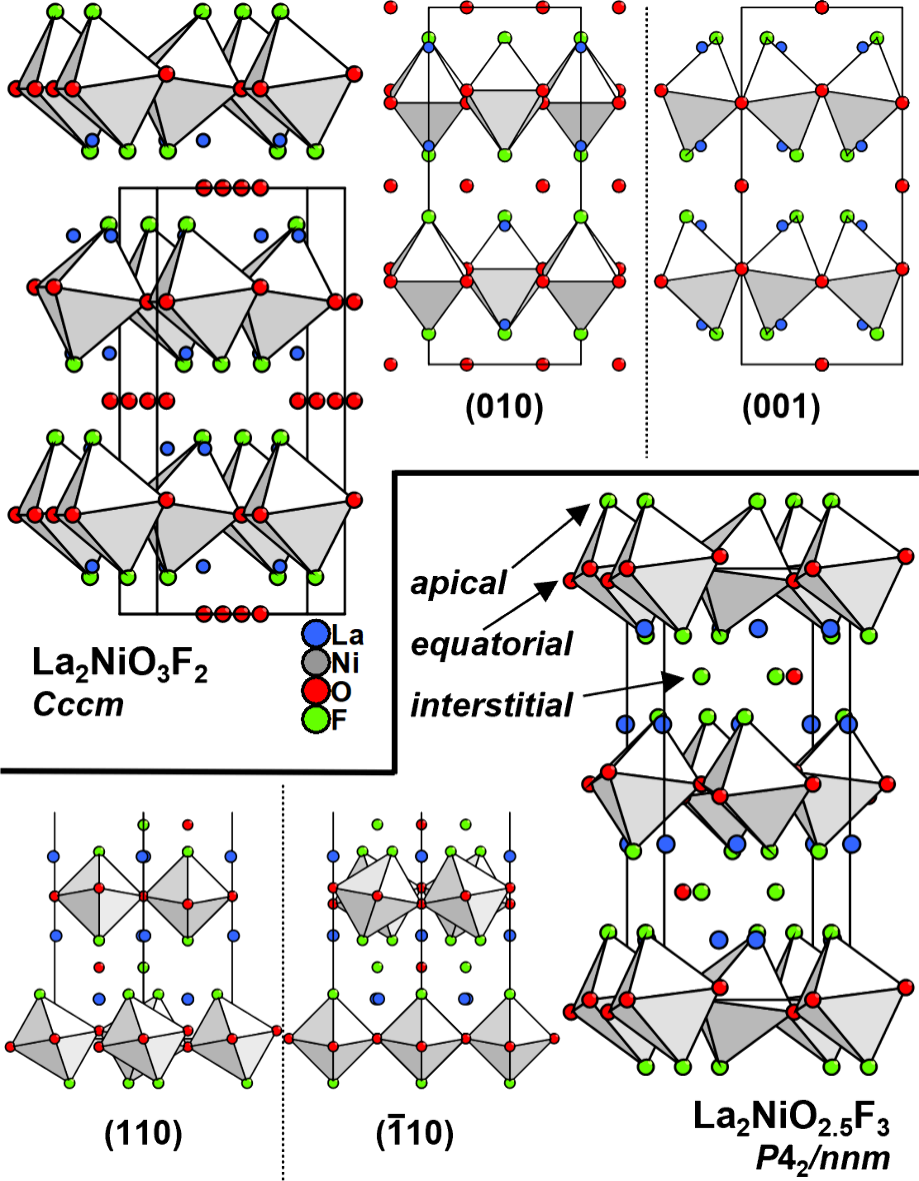
Crystal structures of 2F-oxyfluoride La_2_NiO_3_F_2_ (top) and 3F-oxyfluoride La_2_NiO_2.5_F_3_ (bottom). Additional views on different (*hkl*) planes are shown for both structures in order to highlight octahedral tilting as well as interlayer occupations. Anion distribution was obtained from bond valence sum calculations for both compounds.

In previous studies, the reaction of La_2_NiO_4_ with PVDF ((CH_2_CF_2_)_*n*_) was found to yield two different oxyfluorides, namely, La_2_NiO_3_F_2_^20^ (in the following denoted as 2F-oxyfluoride) and La_2_NiO_2.5_F_3_^14^ (3F-oxyfluoride), depending on the used amount of PVDF. Both oxyfluorides exhibit highly different distortion variants of the tetragonal (*I*4/*mmm*) K_2_NiF_4_ archetype structure, resulting from deviating anion orderings (shown in Figure 1). The 2F-oxyfluoride crystallizes in space group *Cccm*(20) with the NiO_4_F_2_ octahedral tilting system *a*^–^*a*^–^*c*^0^/*a*^–^*a*^–^*c*^0^ (in modified Glazer’s notation^21,22^) caused by F-substitution of apical octahedral position together with the formation of O^2–^ channels around half of the possible anion positions in the interstital layer. Instead, La_2_NiO_2.5_F_3_ crystallizes in space group *P*4_2_/*nnm* with the octahedral tilting scheme *a*^–^*b*^0^*c*^0^/*b*^0^*a*^–^*c*^0^, resulting in a layer-wise rotation of the NiO_4_F_2_ octahedra around one Ni–O–Ni axis. Here, 3/4 of the anion positions in the interlayer are occupied as derived from neutron diffraction experiments and theoretical calculations.^14^ Within this interlayer, two of the four anionic positions are occupied with F^–^, and one O^2–^ is located on the third interstitial anion position, while the fourth remains vacant.

The 2F-compound is easily obtained from reacting mixtures of La_2_NiO_4_ with PVDF (ratio 1:1 oxide to CH_2_CF_2_) at 370 °C for 24 h,^20^ and the system tolerates deviations in the reaction temperature (up to 410 °C) as well as in the dwell times. The 3F-compound, on the other hand, was found to be metastable at the applied reaction temperature (370 °C), resulting in a starting decomposition when reaction times exceeded 15 h, which is only 2 h after the formation is completed.^14^ This is why precise monitoring of the reaction temperature and time is needed for the synthesis of this compound.

In this work, we discuss the results of extensive *in situ* XRD experiments performed on the fluorination reaction in order to gain insight into the formation of both oxyfluorides. The results are supported by *in situ* and *ex situ* neutron powder diffraction experiments as well as ^19^F MAS NMR experiments, yielding information on the different F-anion sites as well as the consumption of PVDF during the reaction. With these experiments, we aim to gain a deeper understanding of the topochemical fluorination reaction itself and of the structure of the reaction intermediates involved in the oxyfluoride formation.

## Experimental Section

### Synthesis

The starting oxide La_2_NiO_4_ was obtained by a citric acid assisted combustion method as reported before.^14^ Stoichiometric amounts of La_2_O_3_ (Merck) (dried at 900 °C for 10 h) and Ni powder were dissolved in ∼50 mL of demineralized water and a few drops of concentrated HNO_3_. After dissolution, citric acid was added in the molar ratio metal ions:citric acid of 1:3 while stirring. By heating the solution to 100 °C on a hot plate, water was evaporated and a citrate gel was obtained, which was further heated at 350 °C until ignition. The obtained powder was subsequently calcined in air at 1050 °C for 6 h. The reaction mixture for the *in situ* experiments was obtained by mixing the oxide with poly(vinylidene fluoride) ((CH_2_CF_2_)_*n*_/PVDF) (Alfa Aesar) in molar ratios of 1:1 and 1:1.5 (oxide:CH_2_CF_2_) using an agate mortar. *Caution*: Heating pure PVDF above its decomposition temperature of 400 °C leads to the release of toxic HF. In contrast, no HF was detected when heating the polymer in the presence of La_2_NiO_4_ or other oxides like CaO.^23^ Instead, CO_2_(g) is released and the oxyfluoride synthesis should be performed in a fume hood.

### Characterization

X-ray diffraction (XRD) patterns were recorded on two diffractometers: (1) A Bruker AXS D8-Advance Bragg–Brentano diffractometer using Cu Kα_1,2_ radiation and a LYNXEYE 1D silicon strip detector (scans were performed in the angular range of 2θ = 10–140° with a data point resolution of 0.01° and 3 s detection time per data point); (2) A STOE STADI MP diffractometer equipped with a MYTHEN2 1K (DECTRIS) silicon strip detector with monochromatic Mo Kα_1_ radiation in film-transmission geometry. Patterns used for structural refinements were averaged from three individual scans in the 2θ range of 5–75° with a data point resolution of 0.015° and 90 min total acquisition time per scan.

High temperature XRD data were also obtained on the STADI MP diffractometer using a HT1 capillary furnace (STOE). Calibration of the internal type S thermocouple was carried out against an external type K thermocouple placed at the sample position inside the capillary. This approach was preferred over the use of thermal lattice expansions or phase transitions, as the same type K thermocouple is also used to check the temperature of the furnaces used for synthesizing larger batches. For *in situ* XRD measurements, samples were filled in capillaries (*d*_ext._ = 0.5 mm), which were open to atmospheric air allowing for a free exchange of the reaction atmosphere. An optimized heating program consists of a ramp (25 K/min) to 300 °C followed by a slower second ramp (5 K/min) to avoid overshooting the desired reaction temperature (300–400 °C). Trials with deviating heating ramps in the range of 5–50 K/min did not result in the observation of different intermediates. Patterns were repeatedly recorded in the angular 2θ range of 9–35° with a 15 min total acquisition time per scan. Sample spinning was employed during the experiments to minimize any potential influence of the sample texture on the obtained diffraction patterns.

Neutron powder diffraction (NPD) data was recorded on three different instruments: (1) The time-of-flight (TOF) diffractometer POWGEN^24^ at the Spallation Neutron Source (SNS) at Oakridge National Laboratory. Here, the powdered sample (∼400 mg) was loaded in a 3 mm diameter cylindrical vanadium container. High resolution data was collected at 300 K in the incident bandwidth center of 2.665 Å for ∼1.5 h of total acquisition time. Data reduction was performed with the MANTID^25^ suite of diffraction utilities. (2) The continuous wavelength (CW) diffractometer Echidna^26^ located at the open light pool reactor (OPAL) operated by the Australian Nuclear Science and Technology Organization (ANSTO). Data was collected for a 1.5 g sample loaded in a 6 mm cylindrical vanadium container with a collection time of approximately 5 h at a wavelength of λ = 2.439 Å. (3) The high intensity CW-diffractometer D20^27^ located at the Institut Laue-Langevin (ILL). This instrument was used for the *in situ* diffraction,^28^ and sample mixtures (∼1 g) were placed in custom-made low background sapphire single crystal cells^29^ which were open to atmospheric air. Heating was realized using two commercially available heat guns, which were positioned at a 120° angle and a distance of approximately 80 mm to the sample (see Figure S1). The temperature of the heated zone was monitored with a pyrometer (pyrosoft). Diffraction patterns were repeatedly recorded with 10 min total acquisition time at a takeoff angle of 120° with a wavelength of λ = 1.880 Å.

FullProf^30^ as well as GSAS II^31^ were used for Rietveld refinements of XRD and NPD data. Instrumental parameters were refined against reference materials (LaB_6_ (STADI MP), Al_2_O_3_ (D8 and ECHIDNA), and Si (D20)). For the TOF data peak shape, parameters were optimized from a preliminary LeBail fit and afterward fixed for the subsequent Rietveld refinements. The background was fitted by interpolation between a set of fixed data points (*ex situ* NPD and XRD data) or a fixed background diffractogram calculated with the pybaselines algorithm, which is implemented in the GSAS II suite (*in situ* XRD and NPD data). Significant preferred orientation effects were excluded for all XRD scans in preliminary refinements, and the March–Dollase parameter was fixed to 1, limiting the number of refinable variables in the final refinement cycle.

To study the fluorine environment in the oxyfluorides, ^19^F Magic Angle Spinning (MAS) NMR experiments were performed on a 1.4 T magnet operated with an Avance II Bruker NMR console running Topspin V2.1 and operating at a frequency of 56.4 MHz. Magic angle sample spinning on the 1.4 T magnet were carried out using a homemade conical stator with the sample packed in 3D printed conical rotors, spinning at approximately 19 kHz.^32^ The chemical shift values refer to CFCl_3_, according to the IUPAC list of reference compounds.^33^ The spectra were analyzed with deconv2Dxy.^34^ The repetition delay was set to 1 s.

## Results and Discussion

### Qualitative Description of the Reaction between La_2_NiO_4_ and PVDF: What Can We Learn from Laboratory *In Situ* X-ray Diffraction Data?

**Figure 2:**
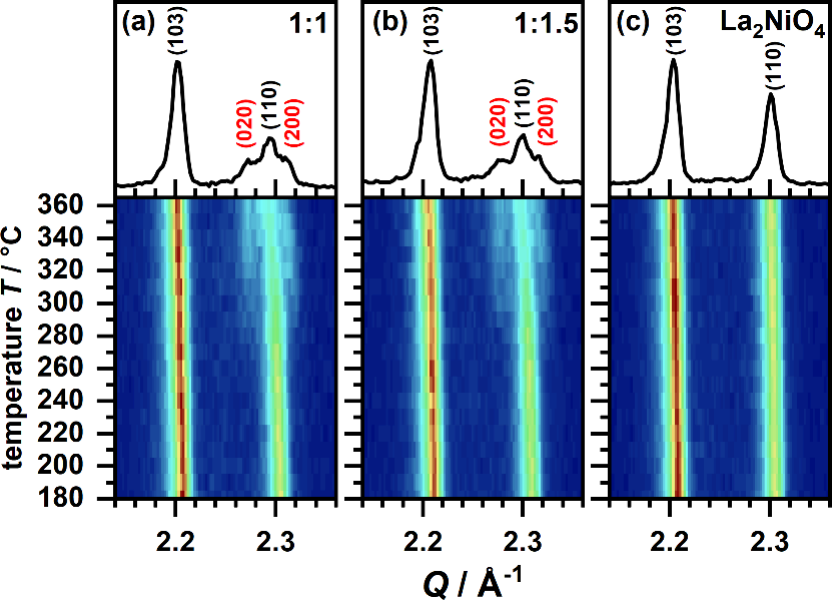
Contour plots in the region of the most prominent reflections of the temperature dependent XRD patterns obtained while heating mixtures of La_2_NiO_4_ with PVDF (CH_2_CF_2_) in the ratios of 1:1 (a) and 1:1.5 (b) and pure La_2_NiO_4_ (c) to 370 °C. The diffraction patterns at 370 °C are additionally plotted in the top part. The diffraction patterns were obtained with 1 min scan duration.

In this first section, we demonstrate the high usefulness of laboratory *in situ* XRD studies for gaining first insights into the complex fluorination pathway of Ruddlesden–Popper oxyfluorides and to identify suitable reaction conditions. To elucidate the differences in the fluorination reaction of La_2_NiO_4_ in dependence of the amount of fluorinated polymer, reactions of two different La_2_NiO_4_/PVDF mixtures (1:1, and 1:1.5) were investigated by temperature and time-resolved X-ray powder diffraction. The use of more reactive soft chemistry precursors with smaller particle size has been proven to be beneficial for obtaining phase pure samples of the targeted oxyfluorides (La_2_NiO_3_F_2_ and La_2_NiO_2.5_F_3_) and all members of the corresponding substitution series La_2_Ni_1–*x*_Cu*_x_*O_3_F_2_,^18^ which is why we used La_2_NiO_4_ obtained from citrate synthesis for all investigations. All samples were taken from the same batch in order to ensure comparability. Samples were contained in capillaries with 0.5 mm outer diameter (wall thickness 0.01 mm), which were open to atmospheric air enabling an unrestricted exchange of the reaction atmosphere. In a first step, reaction mixtures of La_2_NiO_4_ and PVDF in the ratio of 1:1 and 1:1.5 (La_2_NiO_4_:CH_2_CF_2_) were heated from room temperature to 370 °C. The resulting temperature dependent XRD patterns are plotted for the most prominent signals as contour plots in Figure 2a,b. All signals are shifted to smaller *Q* values while heating due to thermal expansion of the unit cell. Starting from *T* ≈ 260 °C, shoulders are found to appear at both sides of the 110 signal of La_2_NiO_4_ (*Q* ≈ 2.3 Å^–1^). This splitting of 110 into 200 and 020 is not observed when pure La_2_NiO_4_ is heated to 370 °C (Figure 2c). Thus, the formation of a first reaction intermediate with orthorhombic unit cell symmetry can be concluded. The fluorination reaction therefore already starts at ∼260 °C. This is significantly below the reaction temperature of 370 °C, which is commonly used in literature and which was also used for the preparation of La_2_NiO_3_F_2_ and La_2_NiO_2.5_F_3_ in previous studies.^7,14^ Interestingly, a very similar observation (reaction starting at 250 °C and 350 °C as preferred reaction temperature) was already reported by Slater in the original article dealing with PVDF as fluorination agent (in this case for the preparation of Sr_2_TiO_3_F_2_ and Ca_2_CuO_2_F_2_)^16^ but has not been mentioned ever since to the best of our knowledge.

After the heating step, isothermal *in situ* XRD experiments were carried out in the temperature range 300−400 °C (Δ*T* = 10 °C) in order to check for reaction intermediates and to find optimal reaction temperatures. Best results in terms of reaction rate and absence of decomposition products were obtained at 380 °C for 2F-oxyfluoride (La_2_NiO_3_F_2_) and 330 °C for 3F-oxyfluoride (La_2_NiO_2.5_F_3_). These temperatures were later used for the synthesis of larger batches of both oxyfluorides, and the obtained reaction parameters were found to translate very well to the synthesis of bulk compounds.

For the following detailed description of the reaction intermediates, the data at 300 °C is used because this comparatively low temperature yields decreased reaction rates (overall reaction duration of ∼40 h), which enables the collection of higher quality X-ray diffraction data (∼15 min per scan) in the range of *Q* = 1 to 5.5 Å^–1^ without sacrificing too much temporal resolution. The 15 min scan time was derived from additional experiments with varying durations (1 min per scan being the shortest), which were performed to exclude the occurrence of any reaction intermediates with faster reaction dynamics. The XRD data of the formation reaction of La_2_NiO_2.5_F_3_ with 1 min scan time is shown in Figure S2. No additional reflections were observed. While 1–5 min time resolution is sufficient to characterize the reactions with the used diffractometer, we intentionally present the 15 min data in order to encourage other authors to perform similar experiments with laboratory diffractometers, which might have a lower performance. All phases whose reflections are observed in the 300 °C data were also found for *in situ* experiments at other reaction temperatures and the diffraction patterns for *T* = 300 °C, 340 °C, 360 °C, and 380 °C are shown in Figure S3. An influence of the reaction temperature on the presence or absence of reaction intermediates can thus be excluded.

**Figure 3:**
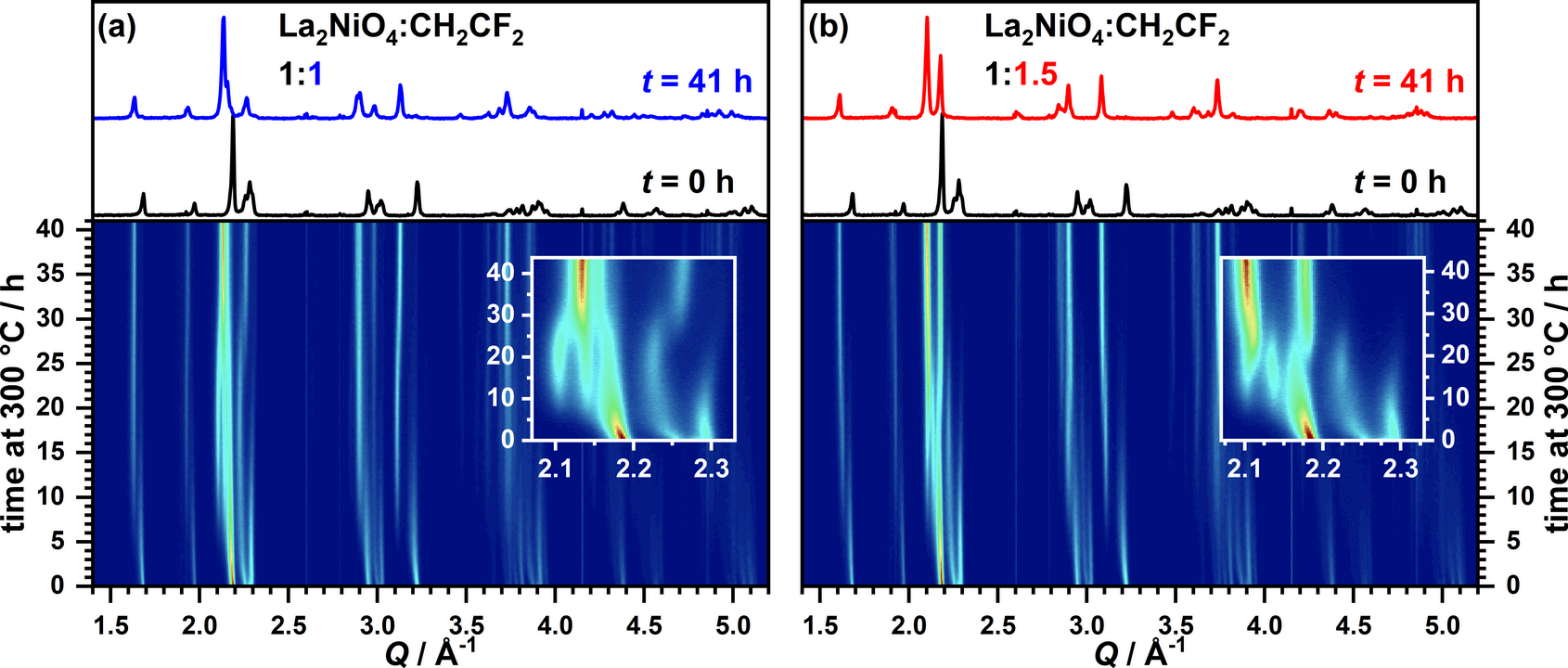
Contour plots of the XRD patterns obtained from time dependent experiments at 300 °C for La_2_NiO_4_:PVDF mixtures with ratios of 1:1 (a) and 1.1.5 (b). The diffraction patterns of the first and last time step are additionally plotted in the top part.

Contour plots of the 300 °C *in situ* XRD data are shown in Figure 3a,b for the two reaction mixtures. The reactions of both La_2_NiO_4_/PVDF mixtures (1:1 and 1:1.5) yield phase pure products (namely, La_2_NiO_3_F_2_*Cccm* and La_2_NiO_2.5_F_3_*P*4_2_/*nnm*) with different diffraction patterns that are additionally shown in Figure 3. Reflections from deviating phases are observed in the course of both reactions (compare the insets in Figure 3 highlighting the evolution of the main reflections), stemming from distinct fluorination steps.

**Figure 4:**
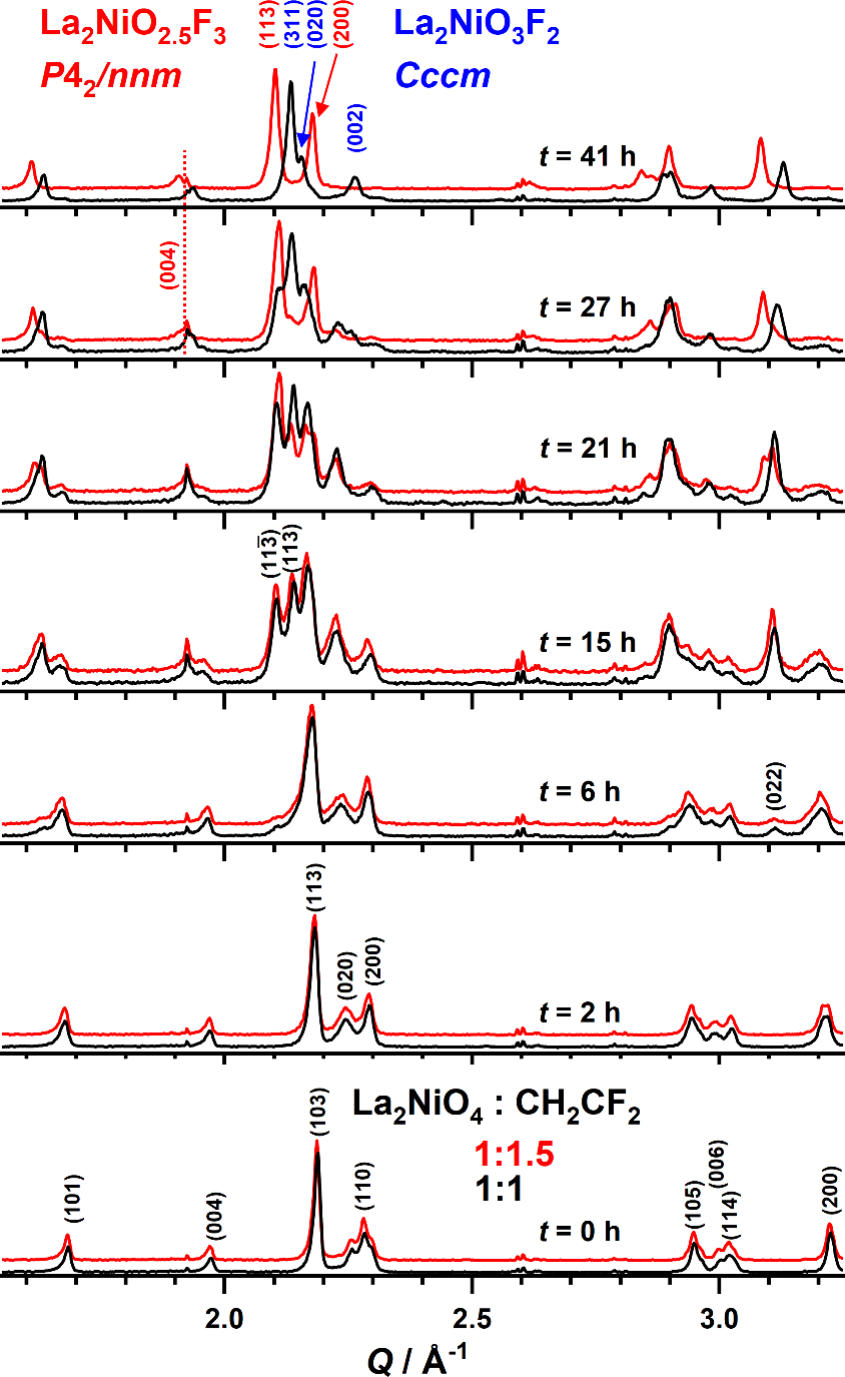
XRD patterns from two isothermal *in situ* experiments at 300 °C for the reactions of La_2_NiO_4_ with PVDF in the ratios of 1:1 (black lines) and 1:1.5 (red lines). The latter are shifted along *y* for better comparability. Miller indices are given for selected peaks only.

Selected XRD patterns of both experiments are plotted in Figure 4 for different reaction durations . This enables a first qualitative assignment of the different patterns to the reaction intermediates. The shoulders, which were indexed as 200 and 020 during heating, clearly belong to a first reaction intermediate (hereafter referred to as **Inter#1**) with orthorhombic lattice. After reaching the maximum reaction temperature, signals of this phase become significantly more intense with increasing duration while a decrease of signal intensity of the initial oxide is observed. For *t* = 2 h, no signals of tetragonal La_2_NiO_4_ are present while the signals of **Inter#1** reach their maximum intensity. In the diffraction patterns at *t* = 2 h and 6 h, a strong broadening of *hkl* signals with *k* contribution like 020 occurs. This broadening increases with *t* and stems from the second reaction intermediate (**Inter#2**). This phase coexists with **Inter#1** and also exhibits orthorhombic unit cell symmetry but with increased orthorhombic deformation as derived from the stronger splitting of 200 and 020. In the *t* = 6 h data, the first signs of a third reaction intermediate (**Inter#3**) are additionally present, most clearly seen as the shoulder on the left side of the 113 main reflection as well as in the appearance of the significantly shifted 022 signal at *Q* = 3.1 Å^–1^. These signals significantly gain intensity with increasing reaction time, and the observed shoulders turn into two clearly separated reflections, which are indexed with 113̅ and 113 in the *t* = 15 h data. **Inter#3** therefore exhibits monoclinic unit cell symmetry. Beginning with the occurrence of **Inter#3**, the observed diffraction patterns for the 1:1 and 1:1.5 ratios clearly deviate from each other for *t* > 15 h due to the formation of the targeted oxyfluorides. La_2_NiO_3_F_2_ forms directly from **Inter#3**, and at *t* = 41 h, only signals belonging to the orthorhombic structure of this compound are present. For the 1:1.5 reaction mixture, signals of a fourth reaction intermediate (**Inter#4**) are found in the *t* = 27 h data. This compound exhibits a similar diffraction pattern as La_2_NiO_2.5_F_3_ (*P*4_2_/*nnm*) and thus shares a similar tetragonal structural distortion. On the other hand, **Inter#4** has a shorter *c*-axis, which is indicated through differences in the positions of reflections with strong *l* contribution like 004, which is highlighted by the dotted line in the *t* = 27 and 41 h data. The 3F-oxyfluoride La_2_NiO_2.5_F_3_ is finally obtained from **Inter#4** after *t* = 41 h. It is remarkable that up to the formation of **Inter#3** both fluorination reactions show qualitatively the same steps, even though the final oxyfluorides exhibit different structures as well as deviating anion contents. It is further notable that the 3F-oxyfluoride is not obtained by progressive fluorination of the 2F-compound and that the excess amount of PVDF clearly needs to be present in the reaction mixture already at the start of the 3F-formation reaction. Further investigations have in fact shown that it is not possible to convert the already produced 2F-oxyfluoride into the 3F-oxyfluoride by reaction with additional PVDF. This approach only results in the formation of LaOF/LaF_3_ mixtures and other decomposition products.

### Structural Description of the Different Reaction Intermediates

In the previous section, we demonstrated that *in situ* XRD experiments are useful for identifying different reaction intermediates based on the presence/absence of their diffraction patterns. The following sections deal with the structural description of the reaction intermediates. A full structural description of oxyfluorides based on XRD data is complicated by the fact that locating light atoms (O/F) in the presence of heavy atoms like La or Ni is challenging due to the very weak contribution of the anionic lattice to the peak intensities. Locating the anionic positions is in principle possible by neutron powder diffraction where O/F atoms have large enough scattering lengths, allowing a robust refinement of the anionic lattice. This is why we obtained *in situ* neutron powder diffraction data sets for both fluorination reactions, as well as *ex situ* ND data for quenched samples. The differentiation of O and F on the anion positions is nevertheless not possible by neutron diffraction alone as their scattering lengths are almost identical (O: 5.803, F: 5.654).^35^ This is why additional methods like ^19^F MAS NMR (*vide infra*) need to be applied to characterize the O/F distribution on the anion sites.

### Inter#1 and Inter#2: The First Two Reaction Intermediates with Orthorhombic Structure

**Figure 5:**
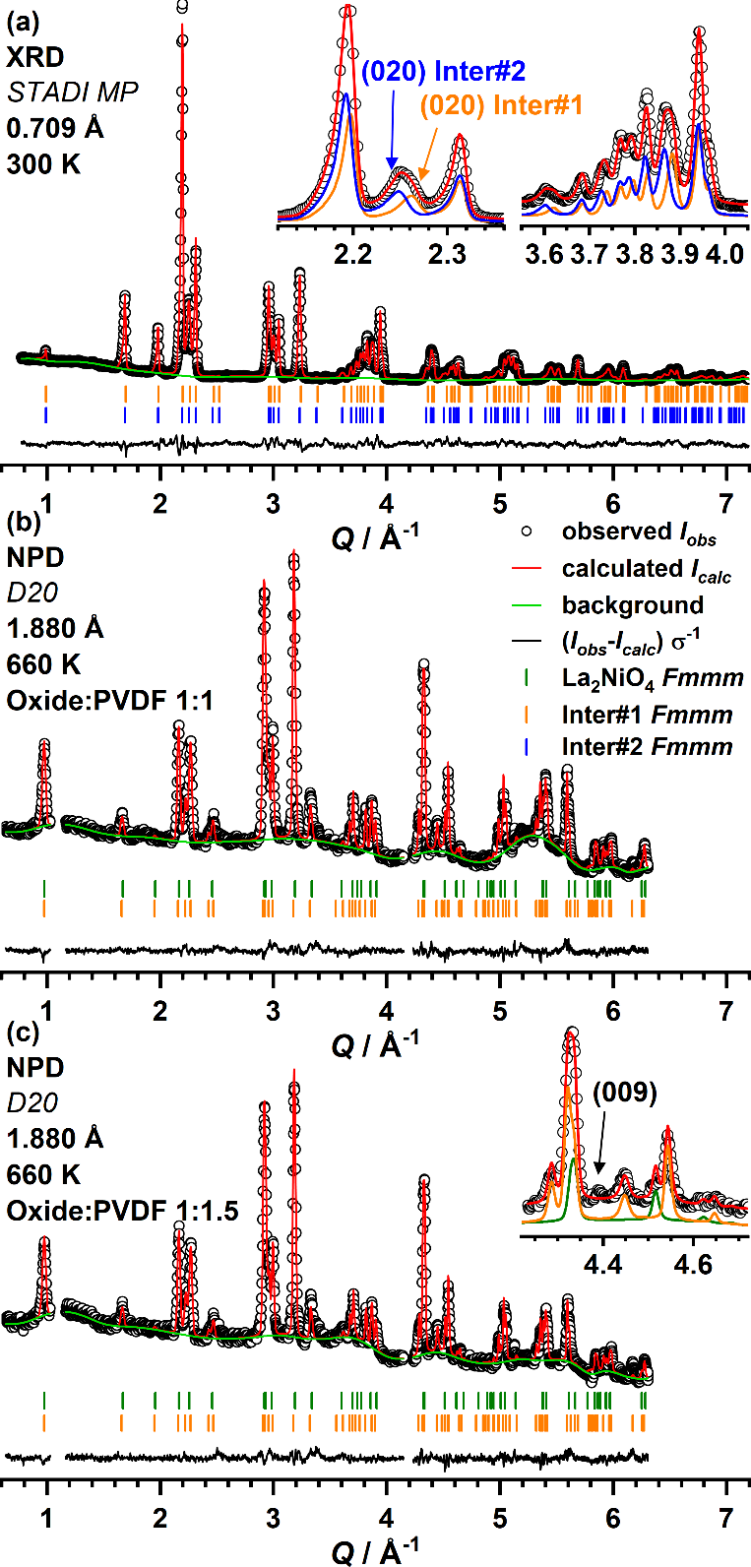
Rietveld plot of the refinements against XRD (a) and *in situ* NPD data (b, c) containing similar amounts of Inter#1 and Inter#2. Both structures were refined in space group *Fmmm*. Simulated diffraction patterns are shown as insets in (a) and (c) to underline the contribution of each phase to the calculated diffraction pattern.

A sample containing similar amounts of Inter#1 and #2 was isolated by quenching ∼100 mg of a 1:1.5 reaction mixture after reaction for 1 h at 370 °C. The X-ray diffraction pattern (plotted in Figure 5a) can be indexed assuming two phases each with orthorhombic unit cell symmetry (Inter#1: *a* = 5.4289(4) Å, *b* = 5.5417(7) Å, *c* = 12.6625(9) Å; Inter#2: *a* = 5.4199(4) Å, *b* = 5.5766(7) Å, *c* = 12.6780(8) Å). Rietveld refinements based on the XRD data were performed using two phases (weight fraction: Inter#1 40%, Inter#2 60%) both with space group *Fmmm* being the highest symmetric orthorhombic sub group of *I*4/*mmm*. The refined parameters are given in Table 1. In contrast to Inter#1, which exhibits no occupation of the interstitial anion sites, Inter#2 shows a partial occupation of the interstitial anion position, as revealed by difference Fourier analysis.

**Table 1:** Structure Parameters Obtained for La_2_NiO_4_ and Both Fluorination Intermediates Inter#1 and Inter#2 from Refinements against *Ex Situ* X-ray Diffraction (XRD) and *In Situ* Neutron Powder Diffraction Data (NPD)

		**Inter#1**		**Inter#2**		**La_2_NiO_4_**			**Inter#1**	
**temperature**		300 K		300 K		660 K	660 K		660 K	660 K
**diffraction method**		XRD		XRD		NPD	NPD		NPD	NPD
**oxide:PVDF**		1:1.5		1:1.5		1:1	1:1.5		1:1	1:1.5

**space group**		*Fmmm*		*Fmmm*		*Fmmm*	*Fmmm*		*Fmmm*	*Fmmm*

***a*/Å**		5.4289(4)		5.4199(4)		5.5607(7)	5.5591(10)		5.5385(3)	5.5371(8)
***b*/Å**		5.5417(7)		5.5766(7)		5.5793(10)	5.5812(11)		5.5659(4)	5.6570(9)
***c*/Å**		12.6625(9)		12.6780(8)		12.8593(14)	12.8555(18)		12.8998(6)	12.8937(16)
**Vol/Å^3^**		380.86(6)		383.19(6)		398.92(7)	398.85(16)		404.36(3)	403.87(15)

**La (8*i*)**	***z*/*c***	0.3616(2)		0.3621(3)		0.3633(8)	0.3650(9)		0.3633(3)	0.3635(4)
**(0,0,*z*)**	***U*_*iso*_/Å^2^**	0.015(4)		0.019(3)		0.0234(6)	0.012(3)		0.0253(15)	0.0384(23)

**Ni (4*a*)**	***U*_*iso*_/Å^2^**	0.017(2)		0.0215(5)		0.027(4)	0.033(4)		0.0306(21)	0.0286(22)
**(0,0,0)**										

**O1@X1 (eq) (8*e*)**	***U*_*iso*_/Å^2^**	0.025(3)		0.034(3)		0.0284(2)	0.017(4)		0.0293(18)	0.0360(26)
**(^1^/_4_,^1^/_4_,0)**										

**O2@X2 (ap)**	**Wyckoff**	8*i*		8*i*		32*p*	32*p*		16*m*	16*m*
	***x*/*a***	0		0		0.057(4)	0.047(5)		0	0
	***y*/*b***	0		0		0.052(5)	0.052(5)		0.0587(11)	0.0538(16)
	***z*/*c***	0.1630(16)		0.1848(13)		0.1748(16)	0.1705(22)		0.1756(7)	0.1761(9)
	***U*_*iso*_/Å^2^**	0.025(3)		0.034(3)		0.028(8)	0.006(8)		0.0198(21)	0.0363(30)
	**s.o.f.^a^**	1		1		0.266(9)	0.259(11)		0.5	0.5

**F1@X3 (int) (8*f*)**	***U*_*iso*_/Å^2^**	−		0.034(3)		−	−		0.0311(4)	0.0311(4)
**(^1^/_4_,^1^/_4_,^1^/_4_)**	**s.o.f.**	0		0.28(2)		0	0		0.21(1)	0.21(1)

***R*_*wp*_/%**		5.75		5.75		4.23	3.37		4.23	3.37
**χ^2^**		4.04		4.04		7.92	5.99		7.92	5.99
**g.o.f.**		2.01		2.01		2.81	2.44		2.81	2.44

a The site occupation factor (s.o.f.) was refined in preliminary runs and fixed to unity if not stated otherwise.

Refinement of the site occupancy factor (s.o.f.) yields a significant occupation of approximately 0.28(2) for this position, resulting in a refined sum formula of La_2_NiO_4_F_0.56_. In a recent preprint, Hancock and Slater report the structure of an oxyfluoride that was obtained by the topochemical fluorination of La_2_NiO_4_ with 0.25 to 0.4 equivalents of PVDF.^36^ The reaction product is also described to crystallize in *Fmmm* (*a* = 5.3678 Å, *b* = 5.6020 Å, *c* = 12.6887 Å) with partial occupation of the anion positions in the LaO interstitial layer. This leads to the assumption that Inter#2 is a partially fluorinated compound with a similar structure. Furthermore, it is highly probable that the orthorhombic unit cell distortion results from tilting induced by the Ni(OF)_6_ octahedra. An ordered octahedral distortion in terms of displacement along *a* or *b* cannot occur for the apical octahedral position X2 at site 8*i* in *Fmmm* due to symmetry limitations (0,0,*z*). Anisotropic refinements of the X2 displacement parameter result in strongly prolate ellipsoids, hinting at tilted octahedra. This question is not addressed by Hancock.

Rietveld plots stemming from independent refinements of two data sets from the *in situ* NPD experiments (targeting the 2F- and the 3F-oxyfluoride) are shown in Figure 5b,c. The contour plots of both experiments are additionally shown in Figure S4. It is found that the signal-to-noise ratio is rather low, mainly because of the high incoherent scattering background resulting from the PVDF hydrogen atoms. First refinements were performed with a model containing two orthorhombic phases each with *Fmmm* symmetry and the obtained structure parameters are listed in Table 1. The first phase exhibits significantly less orthorhombic distortion compared to the XRD data and was assigned to an orthorhombic distorted version of the starting oxide La_2_NiO_4_ without interstitial oxygen occupation. In contrast, a tetragonal (*I*4/*mmm*) unit cell was obtained from the room temperature NPD data in the same experiment with **∼10%** occupation of the interstitial oxygen position (4*d*). This points to an oxygen loss as the first reaction step, which is a hint to a slightly reductive nature of the fluorination with PVDF. Additionally, a displacement of the apical octahedral atoms from (0,0,0.17) to (0.06,0.06,0.17) is obtained from the refinements, which is in concordance with previous descriptions of the orthorhombic structure of La_2_NiO_4_.^37,38^ As the second phase, intermediate Inter#1 is identified from the neutron powder diffraction data (weight fraction: oxide 30%, Inter#1 70%). The same strong anisotropic displacement of the apical atoms of the Ni(O.F)_6_ octahedra along one axis as found in the refinement based on XRD data, pointing to a layer-wise tilting of the octahedra. Such tilting is usually realized in space group *Cmca* (*Bmab* in the coordinate system of *Fmmm*), for example, in La_2_CuO_4_^39^ or in the high temperature modification of La_2_NiO_4._^40^ More recently, a different tilting scheme, with layer-wise opposite tilting of the octahedra was found for La_2_NiO_3_F_2_^20^ as well as strongly defluorinated compounds La_2_NiO_3_F_1.39_, and La_2_NiO_3_F_1.08_,^7^ all with spacegroup *Cccm* (*Bbmb* in the coordinate system of *Fmmm*). Especially, the latter compounds possess a high similarity to the reaction intermediates, which are observed here. Refinements in *Cmca* and *Cccm* did not result in significantly better fits, which is why we stuck to *Fmmm* with less refinable parameters. The displacement of the apical octahedra positions was modeled by introducing a split apical position (0,0,*z*) → (0,∼0.05,*z*). The reaction time dependent evolution of Inter#1 signals, on the other hand, also includes the appearance of a weak signal, which could be indexed as 009 (in the coordinate system of *Fmmm*, marked in Figure 5c). The presence of this peak demands a *C*-centered or primitive unit cell of Inter#1, which we were not able to extract from the present data due to the lack of additional well resolved reflections, indicating the preliminary character of the current structure refinement. Signals of the second phase Inter#2 are also present in the NPD data, especially for longer dwell times. These signals are unfortunately very weak, and their time dependent appearance mostly coincides with the increasing signals of the third intermediate Inter#3, resulting in an under-determination, i.e., too many refinable parameters. This is why we were not able to gain any further information on the structure of this compound and the structural description of Inter#2 is restricted to the results obtained from XRD.

### Inter#3: The Third Reaction Intermediate with Monoclinic Structure

**Figure 6:**
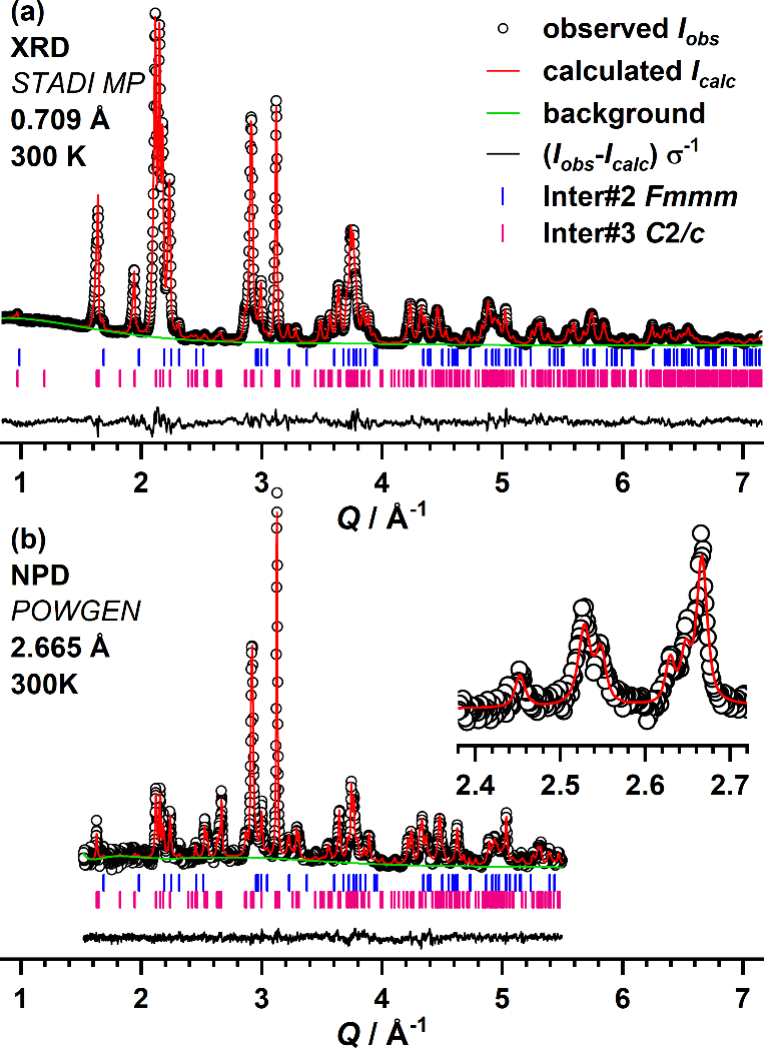
Rietveld plots of the structural refinement of Inter#3 carried out as joint refinement of XRD (a) and TOF-NPD (b) data.

As a third intermediate (Inter#3), a monoclinic phase was identified. The sample containing high amounts of Inter#3 was obtained from quenching a La_2_NiO_4_:PVDF mixture (1:1.5) after a reaction time of 3.5 h at 370 °C. The crystal structure of this phase was refined against combined X-ray and TOF neutron powder diffraction patterns. The obtained structural parameters are listed in Table 2, and the difference plots (XRD and NPD) are shown in Figure 6. As for the previous refinements, reflections of the predecessor phase, in this case Inter#2, are also present with weak intensities. This phase was therefore included in the refinement applying the previously described orthorhombic structure model with space group *Fmmm* (weight fraction: Inter#2 10%, Inter#3 90%). For Inter#3 *C*2/*m*, the highest symmetric *translationengleiche* monoclinic subgroup of *Fmmm* (Inter#1) was used as a structural model. This space group was chosen under the assumption of a progressive symmetry reduction with increasing degree of fluorination. As further candidates, space groups *C*2/*c* and *P*2/*n* were chosen, each representing a *translationengleiche* maximal subgroup of one of the two fully fluorinated final oxyfluorides crystallizing in *Cccm* (2F) and *P*4_2_/*nnm* (3F), respectively. The refinement in *C*2/*m* was least successful, but fits in *C*2/*c* and *P*2/*n* gave similarly good results. The refinement in the lower symmetric space group *P*2/*n* gave no significant reduction of *R*_*wp*_ or χ^2^, despite more refinable parameters. Additionally, no unindexed reflections, which are systematically extinct in a *C*-centered lattice, were observed. This is why *C*2/*c* is probably the correct space group for Inter#3. The assignment of O^2−^ and F^−^ to the individual sites was assumed to be the same as in La_2_NiO_3_F_2_ with O^2–^ occupying the equatorial and interstitial sites and with apical F^–^ occupation. Signals of Inter#3 were also found in the *in situ* NPD data, and two diffraction patterns containing comparable amounts of this intermediate are additionally shown in Figure S5. Here, first refinements yield similar structural parameters for both reaction mixtures, which are also shown in Table 2. It is concluded that this intermediate has the same overall structure, independent of the targeted oxyfluoride stoichiometry. In a recent article, a very similar compound with the composition La_2_NiO_3_F_1.93_ is described in space group *C*2/*c*.^7^ This compound was isolated as the first defluorination product of La_2_NiO_3_F_2_, which is obtained from the reaction with 0.25 equiv of NaH. When the atomic distances of Inter#3 and La_2_NiO_3_F_1.93_ are compared (Table 3), differences between both compounds become evident. In particular, the apical octahedral Ni−X2 distance is significantly larger in Inter#3 (2.167 Å La_2_NiO_3_F_1.93_ vs 2.214 Å Inter#3), indicating more stretched octahedra in Inter#3.

**Table 2:** Structure Parameters of the Third Fluorination Intermediate Inter#3 from Joint Refinements against *ex situ* XRD and NPD Data and Refinements of *in situ* Neutron Powder Diffraction Data^a^

		**Inter#3**		
**temperature**		300 K	660 K	660 K
**diffraction method**		XRD + NPD	NPD	NPD
**oxide:PVDF**		1:1.5	1:1	1:1.5

**space group**		*C*2/*c*	*C*2/*c*	*C*2/*c*

***a*/Å**		12.9143(4)	13.1549(15)	13.1209(29)
***b*/Å**		5.7567(2)	5.8611(7)	5.8575(14)
***c*/Å**		5.6126(2)	5.6789(6)	5.6932(13)
**β/°**		91.31(2)	91.13(1)	91.18(2)
**Vol/Å^3^**		417.15(2)	437.77(6)	437.46(12)

**La (8*f*)**	***x*/*a***	0.1120(8)	0.1087(5)	0.1106(9)
**(*x,y,z*)**	***y*/*b***	0.2492(5)	0.2455(17)	0.2366(29)
	***z*/*c***	0.5213(2)	0.5254(14)	0.5257(23)
	***U*_*iso*_/Å^2^**	0.0098(3)	0.0295(19)	0.031(4)

**Ni (4*c*)**	***U*_*iso*_/Å^2^**	0.0102(5)	0.046(3)	0.027(5)
**(^1^/_4_,^1^/_4_,0)**				

**O1@X1 (eq) (8*f*)**	***x*/*a***	0.2310(4)	0.2357(8)	0.2279(15)
**(*x,y,z*)**	***y*/*b***	0.0250(2)	0.0038(30)	0.018(5)
	***z*/*c***	0.2726(1)	0.2643(21)	0.266(5)
	***U*_*iso*_/Å^2^**	0.0058(2)	0.0152(28)	0.046(3)

**F1@X2 (ap) (8*f*)**	***x*/*a***	0.4149(4)	0.4113(10)	0.4137(17)
**(*x,y,z*)**	***y*/*b***	0.1523(7)	0.1609(16)	0.1584(29)
	***z*/*c***	0.0423(1)	0.0164(27)	0.034(5)
	***U*_*iso*_/Å^2^**	0.0058(2)	0.0414(1)	0.046(3)
	**s.o.f.**	0.944(9)	1	1

**O2/F2@X3 (int) (4*e*)**	***y*/*b***	0.0146(2)	–0.018(4)	0.000(6)
**(0,*y*,^1^/_4_)**	***U*_*iso*_/Å^2^**	0.0058(2)	0.0414(1)	0.046(3)
	**s.o.f.**	1	0.95(3)	1

***R*_*wp*_/%**		2.86	5.04	3.96
**χ^2^**		6.87	6.78	6.54
**g.o.f.**		2.62	2.60	2.56

a The site occupation factor (s.o.f.) was refined in preliminary runs and fixed to unity if not stated otherwise.

**Table 3:** Selected Atomic Distances for La_2_NiO_4_, Inter#3, La_2_NiO_3_F_1.93_, La_2_NiO_3_F_2_, and La_2_NiO_2.5_F_3_

	**atomic distance/Å**				
	**La_2_NiO_4_**(41)	**Inter#3**	**La_2_NiO_3_F_1.93_**(7)	**La_2_NiO_3_F_2_**(20)	**La_2_NiO_2.5_F_3_**(14)
**La−X1 (eq)**	2.610 (×4)	2.466(7)	2.281	2.539 (×2)	2.442
		2.595(3)	2.750	2.813 (×2)	2.715 (×2)
		2.650(7)	2.762		3.005
		3.079(7)	3.005		

**La−X2 (ap)**	2.329	2.373(5)	2.367	2.356	2.529 (×2)
	2.779 (×4)	2.537(6)	2.680	2.602	2.758
		2.613(6)	2.837	2.809 (×2)	
		3.234(6)	2.924		

**La−X3 (int)**	−	2.476(5)	2.324	2.472 (×2)	2.435
		2.481(5)	2.634		2.491 (×2)

**Ni−X1 (eq)**	1.934 (×4)	2.029(8) (×2)	2.024 (×2)	2.008 (×4)	2.026 (×2)
		2.039(8) (×2)	2.063 (×2)		2.056 (×2)

**Ni−X2 (ap)**	2.242 (×4)	2.214(7) (×2)	2.167 (×2)	2.160 (×2)	2.127 (×2)

An increased octahedral stretching of La_2_NiO_3_F_1.93_ compared to the 2F-oxyfluoride is interpreted by Wissel et al.^7^ as an increase of Jahn–Teller active species (in this case Ni^+^) in the compound. A significant amount of nickel in the oxidation state +1 would indicate a reductive character of the fluorination reaction with PVDF. To check for Ni^+^ in Inter#3, magnetization measurements were carried out and the χ vs *T* curves are depicted in Figure S6 in comparison to data obtained for La_2_NiO_2.5_F_3_ and La_2_NiO_2_F_3_. No Curie–Weiss behavior is observed above the transition temperature of 200 K, and therefore, no conclusions can be drawn about the nickel oxidation state from the paramagnetic moment. It has to be emphasized that stretched octahedra are almost always found for RP compounds, and the reason is often attributed to Jahn–Teller distortions. This explanation does not apply for the lanthanum nickelates as Ni^2+^ is not Jahn–Teller active. A more likely reason for the differences in the apical Ni−X2 distances is found in the local environment of La. The corresponding La−X1, −X2, and −X3 distances are listed in Table 3 in comparison to La_2_NiO_4_, La_2_NiO_3_F_2_, and La_2_NiO_2.5_F_3_. The Ni−X2 distance decreases with increasing interlayer occupation, i.e., the coordination number of La. The La−X distances, on the other hand, increase. This is expected considering the bond valence sum (BVS) of +3 for La: with an increasing number of bonds, the individual bond lengths have to increase in order to maintain a constant BVS. Increased La−X values are in principle also expected when cation vacancies exist on the La position. This can be excluded for the present structures as the refinement of the s.o.f. gave no hint for such vacancies. An increased Ni−X2 distance can therefore be interpreted as the sign of a partially unoccupied interstitial position or the exchange of O^2–^ with F^–^ on a fully occupied interlayer position, which would also lower the BVS and, therefore, increase the Ni−X2 distance. With an almost fully occupied interstitial position derived from the refinements, a partial F^–^ interlayer occupation can be deduced. This meets the expectation as the product was obtained by quenching of a 1:1.5 reaction mixture which yields La_2_NiO_2.5_F_3_ with partial F^–^ interlayer occupation as previously reported.^14^

### Inter#4: The Fourth Reaction Intermediate with Tetragonal Structure

**Figure 7:**
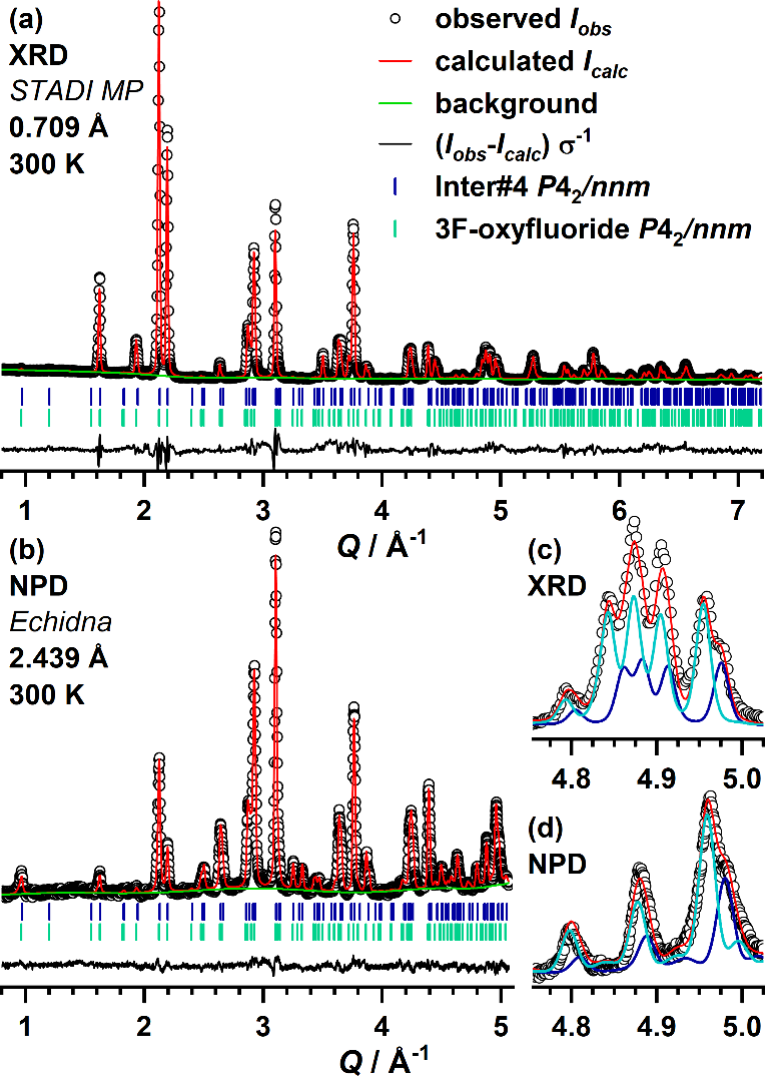
Rietveld plots of the structural refinement of Inter#4 carried out as joint refinement of XRD (a) and CW-NPD (b) data. The calculated patterns of both phases in the refined ratio are plotted in the enlarged region of the XRD (c) and NPD (d) data, highlighting the amount of both phases in the sample.

A sample containing sufficiently large amounts of Inter#4 together with the final 3F-oxyfluoride was obtained by quenching a 1:1.5 reaction mixture after 5.5 h at 370 °C. The structure of Inter#4 was refined based on *ex situ* XRD and NPD measurements. The Rietveld plots are shown in Figure 7, and the structure parameters are listed in Table 4. Here, the simultaneous presence of two phases in the space group *P*4_2_/*nnm* could be confirmed. The sample contains about 67 wt% of the 3F-oxyfluoride and 33 wt% of Inter#4 with a significantly shortened *c*-axis compared to the 3F-phase (12.930 Å vs 13.004 Å corresponding to ∼−0.57%). The *a*-axes of both phases on the other hand are nearly identical with a difference of ∼0.01 Å/0.17% (5.726 and 5.716 Å, 3F vs Inter#4). The anionic positions were assigned analog to the 3F-oxyfluoride due to the strong structural similarity. The refinement of the occupation numbers of the anion positions reveals a less populated 2*b* interstitial site (∼80%) where oxygen is located in the 3F-oxyfluoride. We therefore assume that the reaction from Inter#3 to Inter#4 takes place via incorporation of oxygen from the atmosphere to the interstitial anion positions. In fact, the quenched sample Inter#3 readily reacts to the3F-oxyfluoride with the formation of Inter#4 as intermediate when annealed in air for 1 h. In contrast, when annealing is performed at the same temperature in a N_2_ atmosphere, no reaction occurs even after 3 days. Our interpretation is further supported by *in situ* XRD experiments, which were performed for both La_2_NiO_4_:PVDF mixtures with control of the reaction atmosphere (not shown here). When the reaction is performed in an N_2_ atmosphere, the reaction time is strongly increased, and the reaction already stops at Inter#1 with no further fluorination. When changing the reaction atmosphere at this point to air, the reaction starts again and progresses through Inter#2, #3, and #4 to the final 3F-oxyfluoride.

**Table 4:** Structure Parameters Obtained for the Fourth Fluorination Intermediate Inter#4 from Joint Refinements against XRD and NPD Data

**Atom**	**Wyckoff**	**s.o.f.**	***x*/*a***	***y*/*b***	***z*/*c***	***U*_*iso*_/Å^2^**
**La**	8*m*	1	–0.0039(8)	–*x*(La)	0.3621(1)	0.024(4)
**Ni**	4*f*	1	0	0	0	0.020(1)
**O1@X1a**	4*d*	1	1/4	1/4	0	0.019(8)
**O2@X1b**	4*g*	1	3/4	1/4	0.0058(23)	0.028(5)
**F1@X2**	8*m*	1	0.0735(10)	–*x*(F1)	0.1632(7)	0.0304(2)
**F2@X3a**	4*c*	0.98(4)	1/4	1/4	1/4	0.0304(2)
**O3@X3b**	2*b*	0.78(7)	3/4	1/4	1/4	0.0304(2)


space group	*a* = 5.7169(3) Å	*c* = 12.9301(6) Å	*V* = 422.59(3) Å^3^
*P*4_2_/*nnm*	*R*_*wp*_ = 6.01	χ^2^(NPD+XRD) = 4.96	g.o.f. = 2.23

### Analysis of the Fluorine Environment by ^19^F MAS NMR

**Figure 8:**
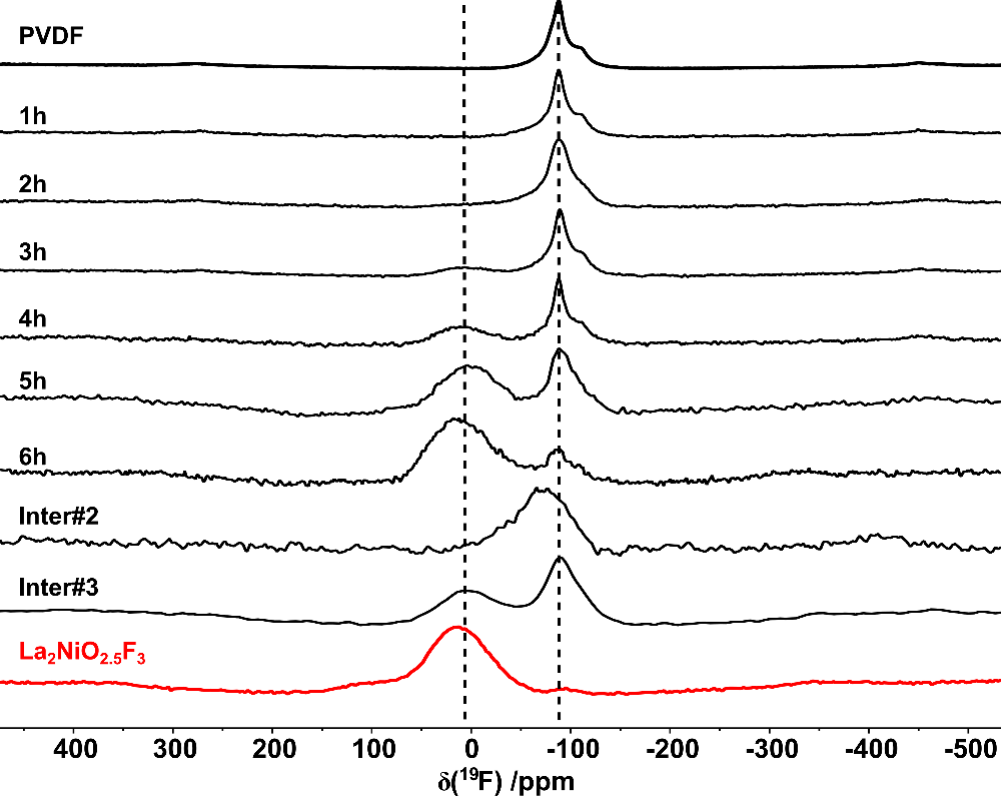
^19^F MAS NMR spectra of selected oxyfluorides obtained from annealing a La_2_NiO_4_:PVDF 1:1.5 mixture at 370 °C for 1 h, 2 h, 3 h, 4 h, 5 h, and 6 h and pure PVDF. Additionally shown are spectra of an Inter#2 sample obtained from the reaction with 30% PVDF, *isolated* Inter#3 obtained from quenching, and the pure 3F-oxyfluoride. The samples were spun at a speed of around 20 kHz in a 1.4 T magnetic field. The lines serve as guides for the eye.

**Figure 9:**
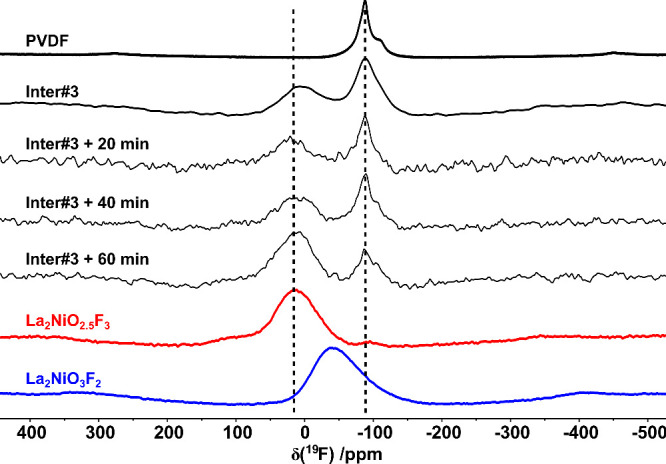
^19^F MAS NMR spectra of Inter#3 annealed at 370 °C for 20, 40, and 60 min. For comparison, the spectra of PVDF, Inter#3, La_2_NiO_2.5_F_3_, and La_2_NiO_3_F_2_ are also shown. The samples were spinning at the speed of around 20 kHz in a 1.4 T magnetic field. The lines serve as guides to the eye.

Up to this point, statements on the fluorine content and positions of the reaction intermediates are made based on occupation numbers and similarities to already published compounds. In order to obtain additional information on the changes in the fluorine environment during the fluorination, ^19^F MAS NMR experiments were performed on selected compounds obtained from quenching a La_2_NiO_4_:PVDF (1:1.5) mixture after different reaction durations at 370 °C in intervals of 1 h. The NMR experiments were performed in a low-field fast-spinning magic angle spinning setup recently developed,^32^ which provides a good suppression of paramagnetic spinning sidebands, while providing semiquantitative information about the PVDF content. The samples contain different fractions of the intermediates as obtained from XRD (the diffraction patterns are shown in Figure S7). The fluorination agent PVDF was measured separately and shows a peak at −88 ppm (Figure 8). This peak is present in all obtained spectra, and its intensity decays with increasing annealing time as PVDF is consumed in the course of the reaction but does not vanish completely. This indicates the presence of some residual PVDF even for the final oxyfluoride, most probably due to the use of a slight excess, which should thus be avoided/tuned for future studies. Data of an orthorhombic compound, which was obtained from reacting a La_2_NiO_4_:PVDF (1:0.3) mixture exhibiting similar unit cell parameters as Inter#2, shows a peak at −70 ppm, most probably stemming from partially incorporation of F^–^ on the LaO interlayer positions. Due to the similarity of this compound to Inter#2, this signal is also expected for the spectra obtained after 1 and 2 h, which contain significant amounts of Inter#1 (60 wt% (1 h), 45 wt% (2 h)) and Inter#2 (40 wt% (1 h), 55 wt% (2 h)). Unfortunately, this signal is not clearly resolved as it overlaps with the strong PVDF signal (−88 ppm), but it can be found as a shoulder at the PVDF signal resulting in a significantly broadened signal for the 2 h sample. With increasing annealing time, a new peak at 12 ppm appears, which is assigned to the phase Inter#3 and is also found in the separate measurements obtained for the quenched Inter#3 sample as shown in Figure 8. Based on Rietveld refinements, the sample obtained after 4 h contains almost 90% Inter#3, while the sample after 5 h contains more than 50% Inter#4. Surprisingly, the NMR spectra of both compounds are qualitatively the same, and both intermediates therefore cannot be distinguished by NMR under these conditions. An additional annealing experiment was performed in finer steps of 20 min at 370 °C starting from the quenched Inter#3 sample, aiming to identify the differences between both phases. The resulting NMR spectra are plotted in Figure 9, and the corresponding XRD patterns can be found in Figure S8. Even though strong changes are observed in the XRD data after 20 and 40 min of annealing, the NMR spectra barely show any difference, especially with respect to the consumption of PVDF. The fact that the signal at 12 ppm does not change after 20 and 40 min of annealing leads to the interpretation that the structural transition (Inter#3 → Inter#4), which is observed by XRD, has to be linked to the oxygen content, most probably due to partial filling of the interstitial positions with oxygen resulting in Inter#4. This interpretation is supported by the fact that further fluorination of Inter#3 and formation of Inter#4 should result in consumption of PVDF, which is not observed (the signal ratio stays the same at 20 and 40 min annealing time). This is also in accordance with the increasing partial interlayer filling found in Inter#4 as discussed above. Only when the sample was annealed for 60 min and La_2_NiO_2.5_F_3_ was formed, the area of the two peaks changed. The reaction of Inter#4 to La_2_NiO_2.5_F_3_ therefore consumes most of the remaining PVDF and results in the formation of the additional peak at 95 ppm, most probably due to F-incorporation into the interstitial layer. Interestingly, the chemical shift of the signal observed for Inter#3 and Inter#4 (12 ppm) strongly deviates from the signal of the pure 2F-compound (additionally shown in Figure 9) with apical F-ion ordering where one signal is found at −44 ppm pointing to a different F-environment of these compounds.^18,20^ This is somewhat surprising, as the structures of Inter#3 and La_2_NiO_3_F_2_ are highly similar. This observation supports the assumption that Inter#3 in the 2F-reaction might exhibit a different structure than Inter#3 occurring in the 3F-reaction, most probably caused by different interstitial anion occupations.

## Summary, Outlook, and Conclusion

**Figure 10:**
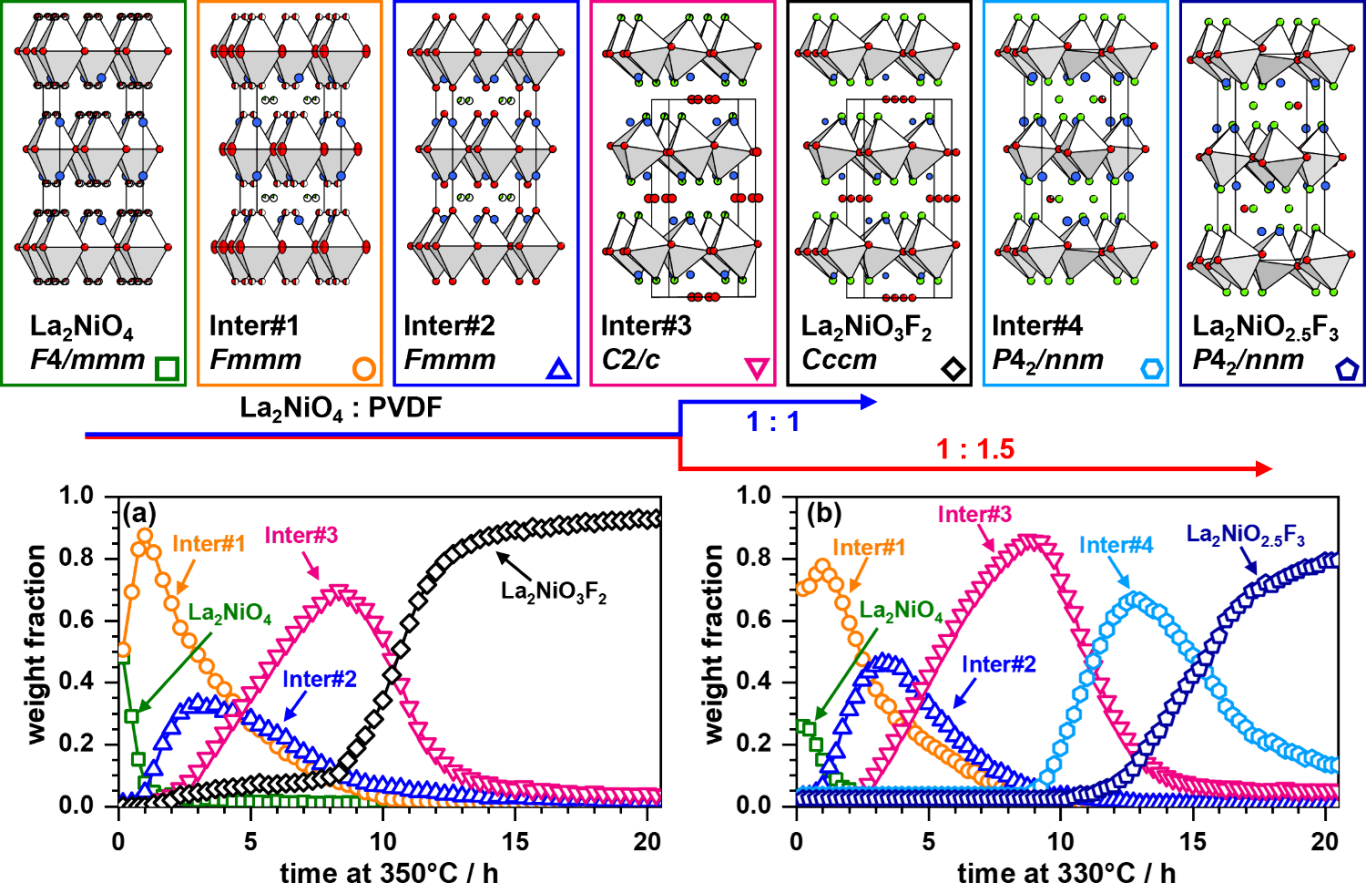
Structural transformations observed during the fluorination reaction of La_2_NiO_4_ with PVDF in the two different ratios 1:1 and 1:1.5 yielding the products La_2_NiO_3_F_2_ and La_2_NiO_2.5_F_3_. The structures were obtained from refinements based on neutron and powder X-ray diffraction data. The phase evolution of both reactions depending on the reaction time as derived from Rietveld refinements of *in situ* XRD data is plotted in (a) and (b) for La_2_NiO_3_F_2_ and La_2_NiO_2.5_F_3_, respectively.

We performed an in-depth study of the fluorination reaction of La_2_NiO_4_ with PVDF in molar ratios of 1:1.5 and 1:1 and successfully identified four reaction intermediates. Structural information for these less fluorinated compounds were derived from XRD, NPD, and ^19^F MAS NMR data. The first three reaction intermediates (Inter#1, Inter#2, and Inter#3) seem to be the same for both reaction mixtures, and they possess a high similarity to partially defluorinated compounds, which were previously found for the reductive defluorination of La_2_NiO_3_F_2_ with NaH.^7^ This highlights the strong flexibility in the anionic compositions of these nickel-based RP oxyfluorides. For the reaction to the 3F-oxyfluoride (1:1.5 mixture), the occurrence of a fourth intermediate was found, which possesses a structure distortion similar to that of the target compound. The complete structural evolution is schematically depicted in Figure 10. Additionally, we provide first weight fraction vs time data, which was extracted from multiphase Rietveld refinements showcasing the phase evolution of all reaction intermediates on a quantitative basis (see Figure 10a,b).

We also demonstrate how ^19^F MAS NMR spectroscopy on a 1.4 T benchtop NMR spectrometer can support such investigations even for paramagnetic oxyfluorides, especially concerning the quantitative description of the consumption of the fluorination reagent, which is not accessible by XRD and for which only qualitative information can be obtained by methods like IR or Raman spectroscopy.

The results of the presented investigations underline the high complexity of topochemical fluorination reactions. A simple laboratory *in situ* X-ray setup is very valuable for synthesis optimization of the low temperature fluorination reactions with PVDF as the fluorine source as well as for gaining in-depth knowledge of the reaction intermediates involved. It should be emphasized that all X-ray investigations were carried out on a commercial laboratory diffractometer and thus no measurement time at large research facilities is necessary for this kind of reaction optimization. We propose that in-house *in situ* XRD investigations should be more routinely used to enable more rational synthesis planning. Ultimately, this will facilitate the synthesis of even less stable oxyfluorides in future studies. We have already successfully synthesized several other oxyfluorides by applying this approach: La_2_CuO_3_F_2_,^18,39^ La_2_CuO_2.5_F_3_, Pr_2_CuO_3_F_2_, Nd_2_CuO_3_F_2_, Nd_2_NiO_3_F_2_, La_2_CoO_3_F_3_, and La_4_Ni_3_O_8_F_4_ to name a few. The description of the structures and properties of these oxyfluorides will be addressed in following contributions.

## Supporting Information Available

Picture of the experimental setup used for the *in situ* NPD experiments; contour plots of *in situ* XRD data obtained for a La_2_NiO_4_:PVDF 1:1.5 mixture with a temporal resolution of 1 min; contour plots of *in situ* XRD data obtained for different isothermal experiments at 300, 340, 360, and 380 °C; contour plots of the NPD data obtained from *in situ* experiments performed for the La_2_NiO_4_:PVDF mixtures; Rietveld plots obtained from the structural refinements of Inter#3 against different data sets; molar susceptibility vs temperature data for Inter#3 in comparison to La_2_NiO_3_F_2_ and La_2_NiO_2.5_F_3_; XRD data of different oxyfluoride samples obtained from annealing and subsequent quenching of a La_2_NiO_4_:PVDF 1:1.5 mixture; XRD data and FTIR spectra for different oxyfluoride samples obtained from annealing Inter#3 (PDF)
