# Supplementary material for: Unveiling the Fluorination Pathway of Ruddlesden–Popper Oxyfluorides: A Comprehensive In Situ X-ray and Neutron Diffraction Study
Source: J Am Chem Soc. 2025 Feb 17;147(11):9739–51. doi: 10.1021/jacs.4c18187 (PMC11926862; doi:10.1021/jacs.4c18187)
Supplement: Supplementary file 1 — ja4c18187_si_001.pdf [file ja4c18187_si_001.pdf]

Unveiling the Fluorination Pathway of Ruddlesden-Popper Oxyfluorides: A Comprehensive *in situ* X-ray and Neutron Diffraction Study

Jonas Jacobs[,1], Andy Bivour[1], Vadim Sikolenko[2], Holger Kohlmann[2], Thomas C. Hansen[3], James R. Hester[4], Ke Xu[5], Jörn Schmedt auf der Günne[5], and Stefan G. Ebbinghaus[1]*

[1] Martin Luther University Halle-Wittenberg, Faculty of Natural Sciences II, Institute of Chemistry, Inorganic Chemistry, Kurt-Mothes-Straße 2,06120, Halle, Germany

[2] Leipzig University, Institute for Inorganic Chemistry and Crystallography, Johannisallee 29, 04103 Leipzig, Germany

[3] Institut Laue-Langevin, 71 avenue des Martyrs, 38000 Grenoble, France

[4] Australian Center for Neutron Scattering, Australian Nuclear Science and Technology Organisation (ANSTO), Locked Bag 2001, Kirrawee DC, NSW 2232, Australia

[5] University of Siegen, Faculty IV: School of Science and Technology, Department of Chemistry and Biology, Inorganic Materials Chemistry, Adolf-Reichwein-Str. 2, 57076, Siegen, Germany

Email: jonas.jacobs@chemie.uni-halle.de

Supporting Information

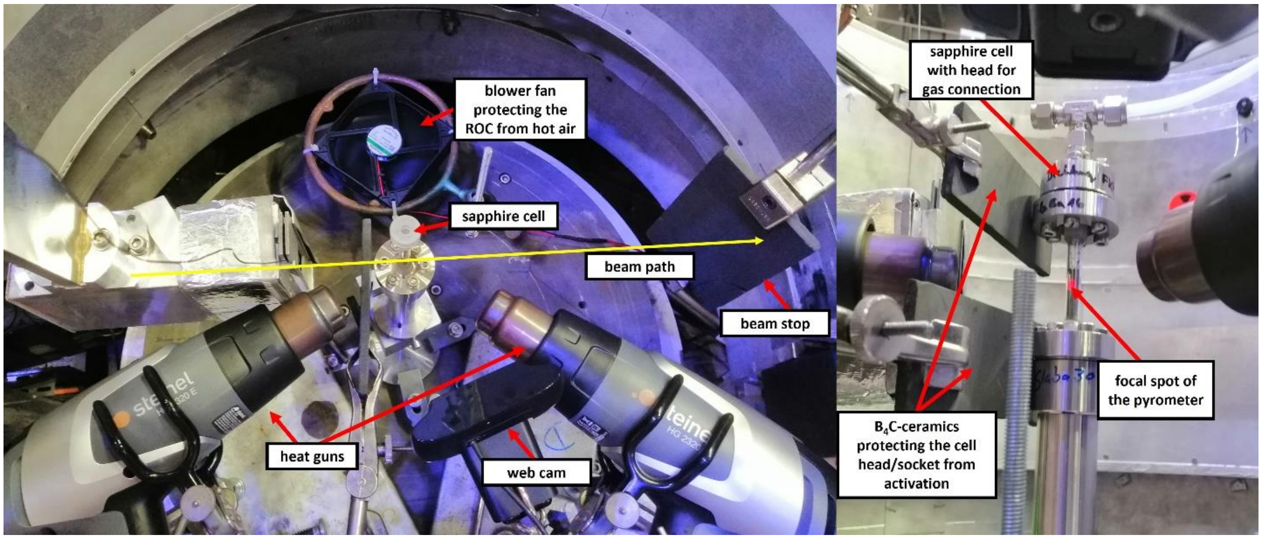

Figure S1:Experimental setup used for the *in situ* NPD experiments at the high flux diffractometer D20 (ILL) consisting of the sapphire cell, two heat guns, a web cam, a pyrometer (not shown), and an upwards pointing blower fan directing the hot air away from the radial oscillating collimator (ROC).

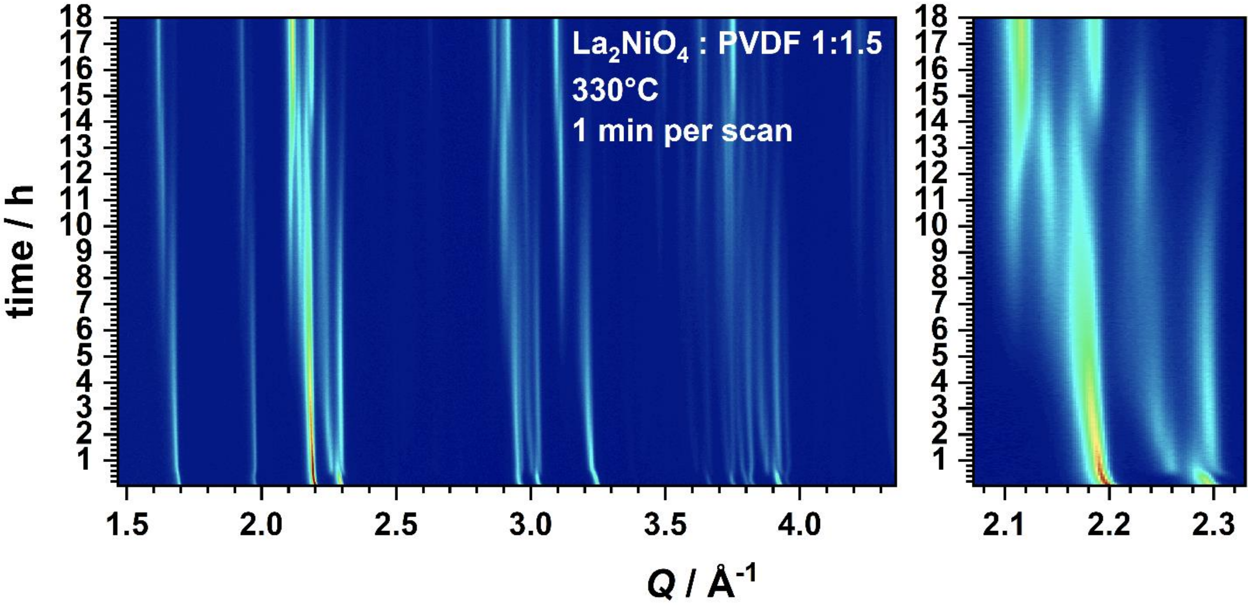

Figure S2: Contour plot of the *in situ* XRD data obtained for the fluorination of La_2NiO_4 with PVDF in a 1:1.5 ratio at 330 °C. The diffraction patterns were obtained with a stationary DECTRIS MYTHEN2 1K detector module centered at 2θ = 18.5°. This allows for acquisition of diffraction patterns in the range of 2θ = 9.5 to 28.5° (Q = 1,47 – 4.35 $Å^{-1}$) without movement of the detector. The temporal resolution was set to 1 min and all signals which are discussed for the 15 min temporal resolution data are also present in the 1 min data. The first 0.5h consist of stepwise heating and T_{max} = 330°C was reached after ~0.5h.

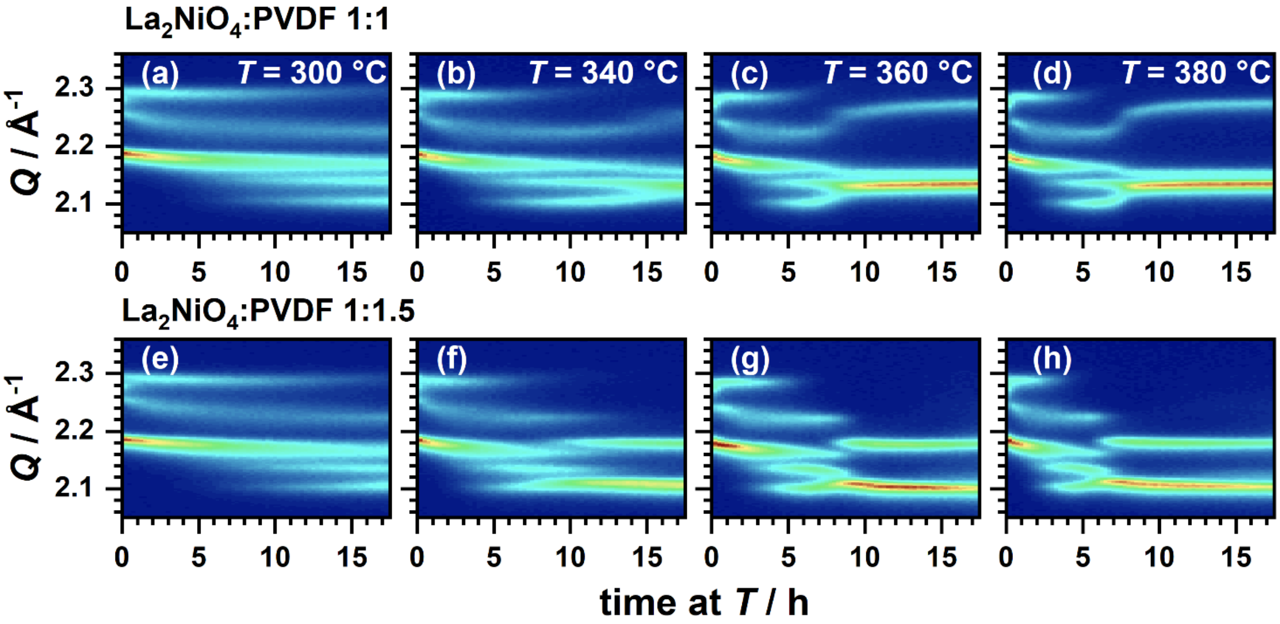

Figure S3: Contour plots of the *in situ* XRD data obtained for performing the fluorination reaction of La_2NiO_4 with PVDF in the ratio 1:1 (a-d) and 1:1.5 (e-f) at different temperatures of 300 °C (a, e), 340 °C (b, f), 360 °C (c, g), and 380 °C (d, h). Only the region of the most prominent reflections is plotted in order to maintain clarity. The observed reflection positions are the same for all temperatures, excluding an influence of the reaction temperature on the presence/absence of certain reaction intermediates.

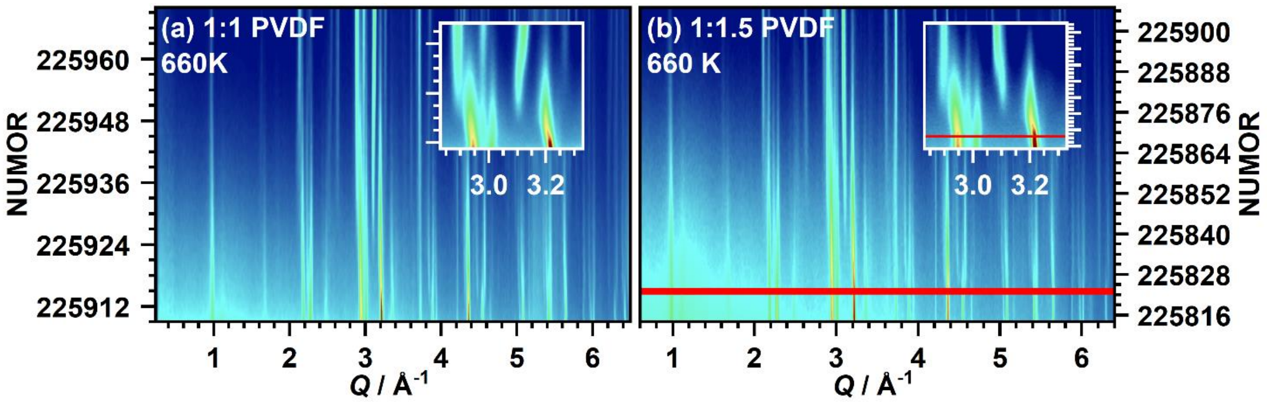

Figure S4: Contour plots of the NPD data obtained from *in situ* experiments performed for two La_2NiO_4:PVDF mixtures with 1:1 (a) and 1:1.5 (b) ratio. Each scan had an acquisition time of 10 min; NUMOR is the internal raw data labelling of the ILL. The red line in (b) indicates a missing scan.

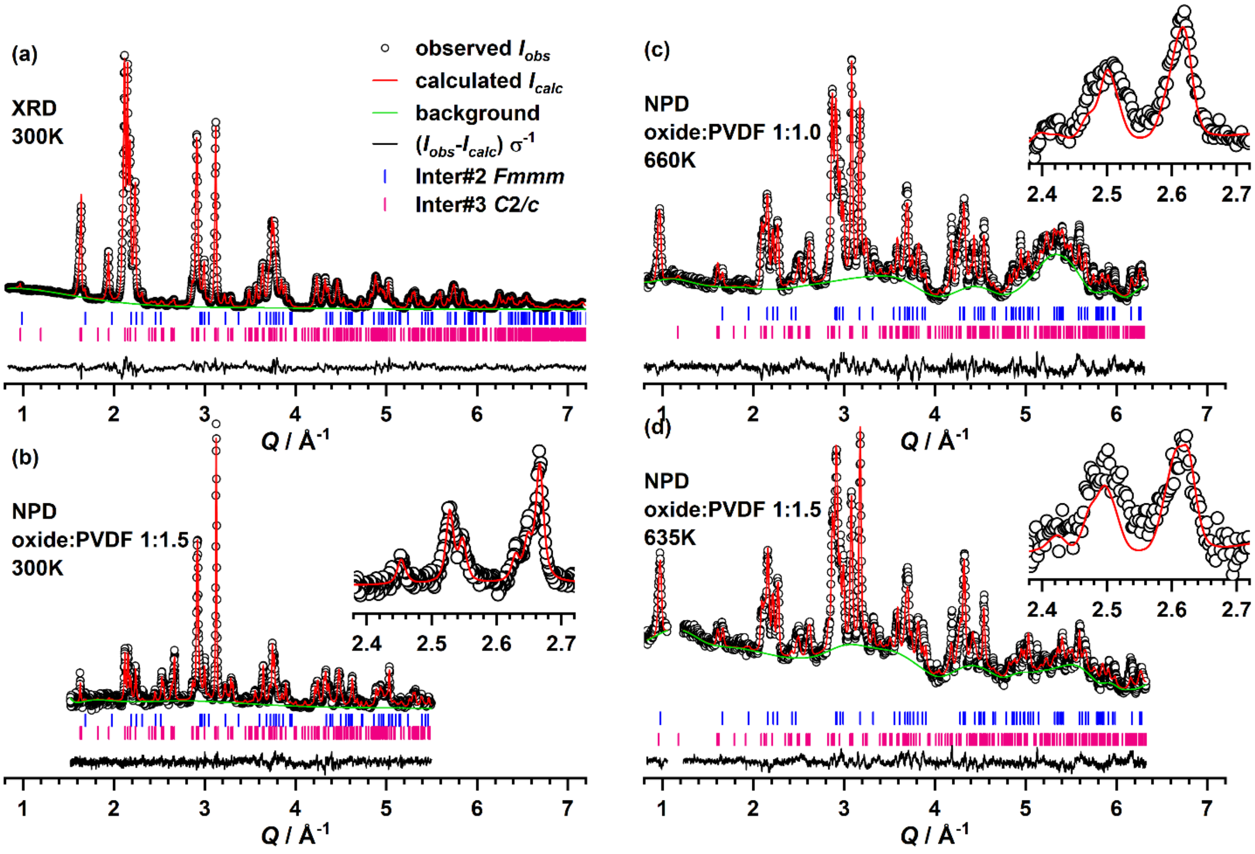

Figure S5: Rietveld plots obtained from the structural refinement of Inter#3 $La_2NiO_{4-c/2}F_c$ against different datasets. Room temperature XRD (a), and NPD (b) data, as well as *in situ* NPD data obtained for a La_2NiO_4:PVDF 1:1 (c) and 1:1.5 (d) mixture. The background was fitted by interpolation between a fixed set of datapoints (*ex situ* NPD and XRD data) or a fixed background diffractogram calculated with the pybaselines algorithm which is implemented in the GSAS II suite (*in situ* XRD and NPD data).

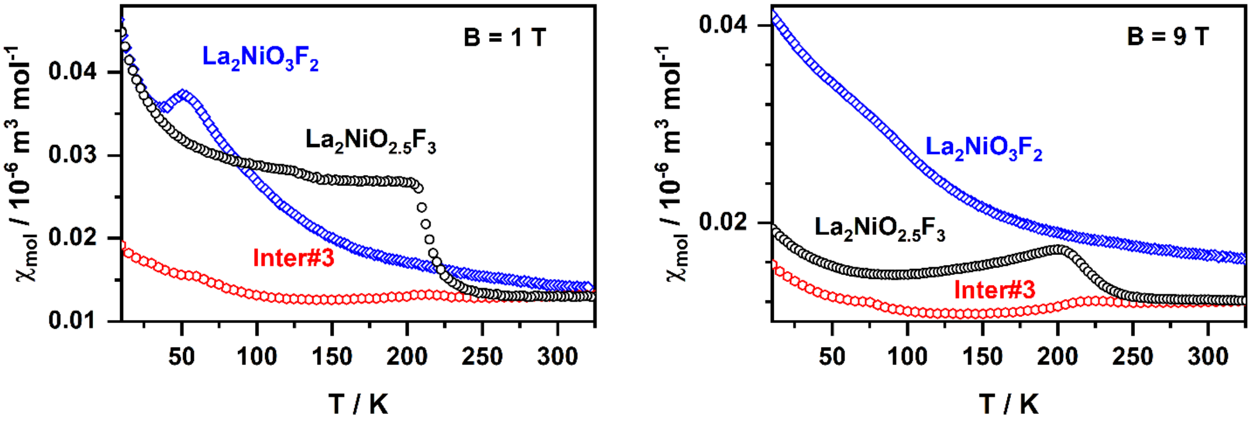

Figure S6: Molar susceptibility vs. temperature at B =1 T and 9 T for Inter#3 in comparison with $La_2NiO_3F_2$ and $La_2NiO_{2.5}F_3$.

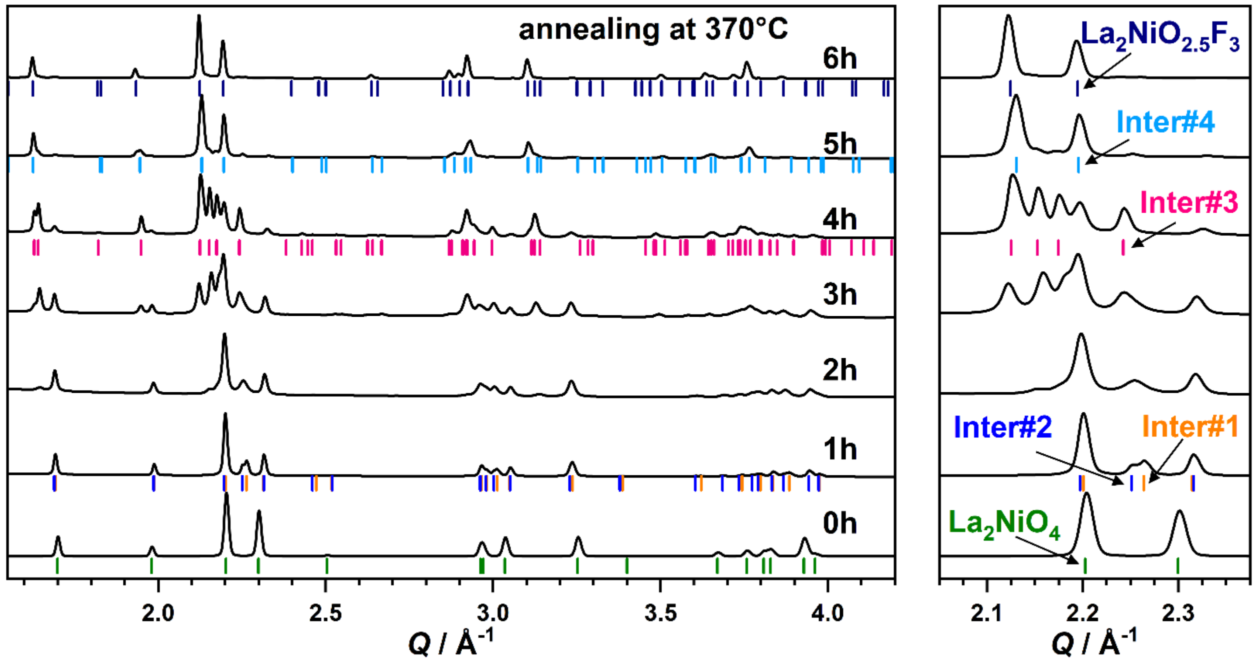

Figure S7: XRD data of different oxyfluoride samples (100 mg each) obtained from annealing and subsequent quenching of a La_2NiO_4:PVDF 1:1.5 mixture for 1 h, 2 h, 3 h, 4 h, 5 h, and 6 h at 370°C. The samples were used in the 19F MAS NMR experiments presented in Figure 8 of the manuscript.

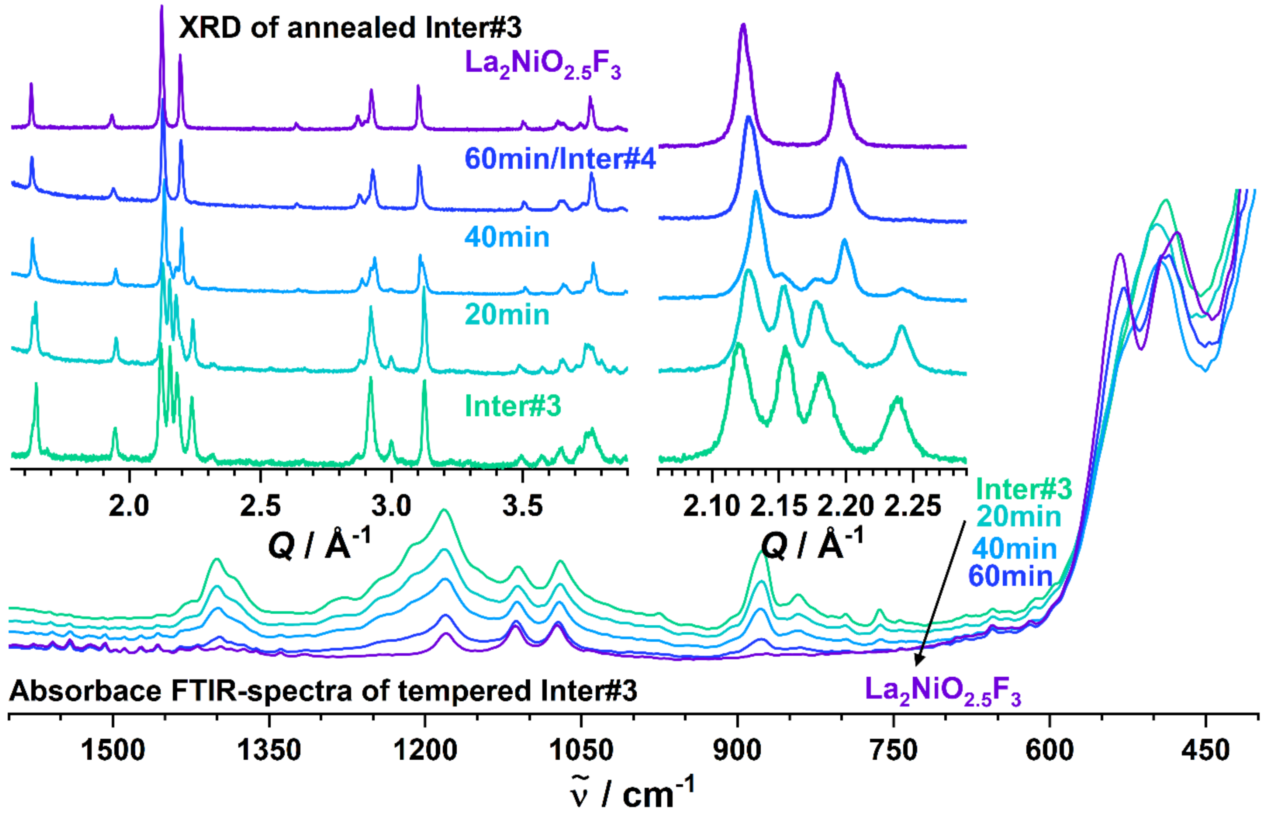

Figure S8: XRD data and FTIR-spectra for different oxyfluoride samples obtained from annealing and subsequent quenching of 100 mg of Inter#3 for 20, 40, and 60 min at 370°C. The samples were used in the ^{19}F MAS NMR experiments presented in Figure 9 of the manuscript.
